# An m^7^G tRNA-derived fragment mediated thermogenic activation of brown adipose tissue through post-transcriptional repression of Uqcr10

**DOI:** 10.1016/j.jbc.2026.113640

**Published:** 2026-10-14

**Authors:** Xue Zhao, Mailin Gan, Xingyu Wang, Yidong Chen, Jiaqi Zhu, Qing Ye, Jincheng Zan, Yuhang Lei, Xiaofeng Zhou, Lei Chen, Ye Zhao, Li Zhu, Yan Wang, Linyuan Shen

**Affiliations:** 1 Farm Animal Germplasm Resources and Biotech Breeding Key Laboratory of Sichuan Province, Sichuan Agricultural University, Chengdu, China; 2 State Key Laboratory of Swine and Poultry Breeding Industry, Sichuan Agricultural University, Chengdu, China; 3 Animal Disease Prevention and Green Development Key Laboratory of Sichuan Province, College of Life Sciences, Sichuan University, Chengdu, China; 4 Key Laboratory of Livestock and Poultry Multi-omics, Ministry of Agriculture and Rural Affairs, College of Animal and Technology, Sichuan Agricultural University, Chengdu, China

**Keywords:** Brown adipose tissue thermogenesis, METTL1, m^7^G modification, tRNA stability, tRF-Lys-CTT, Uqcr10

## Abstract

Obesity, diabetes, and related metabolic disorders have become global epidemics driven mainly by an imbalance between energy intake and energy expenditure. Brown adipose tissue (BAT), the principal site of non-shivering thermogenesis, can be activated to mitigate obesity and its metabolic sequelae. RNA modification is an important regulator of obesity. The N7-methylguanosine (m^7^G) modification broadly influences RNA stability, enhances translational efficiency, and helps determine cell fate. However, the specific mechanisms by which m^7^G regulates adipose thermogenesis remain unclear. Using untargeted metabolomics, we found that cold exposure activates thermogenic metabolism in BAT and that 7-Methylguanosine is elevated in thermogenically activated BAT. METTL1-mediated m^7^G modification correlates positively with adipose thermogenic activation. Loss of METTL1 reduces m^7^G levels and impairs adipocyte thermogenic capacity. At the same time, METTL1 deficiency alters tRNA cleavage patterns and generates regulatory tRNA-derived fragments that modulate gene expression. Overexpression of tRF-Lys-CTT, which derives from an m^7^G-modified tRNA, inhibits brown adipocyte differentiation and thermogenic activation; conversely, inhibiting tRF-Lys-CTT produces the opposite effects. Mechanistically, tRF-Lys-CTT associates with AGO2 to form a functional RISC complex that binds the 3′UTR of *Uqcr10* mRNA and directly represses *Uqcr10* post-transcriptionally. Under basal conditions, elevated tRF-Lys-CTT may constrain mitochondrial function by repressing Uqcr10 expression; upon cold stimulation, downregulation of tRF-Lys-CTT relieves this repression and promotes respiratory chain supercomplex assembly and UCP1-mediated thermogenesis. In summary, our study indicates that tRF-Lys-CTT functions as a post-transcriptional regulator of metabolism that suppresses thermogenesis and provides new insights into the regulatory relationship among the METTL1/m^7^G/tRF axis and thermogenic control.

Treating obesity and its metabolic complications remains a major clinical challenge. These disorders stem largely from a sustained energy imbalance in which intake exceeds expenditure. As a key regulator of whole-body energy metabolism, adipose tissue maintains energy homeostasis and coordinates systemic metabolic responses. Among adipose depots, brown adipose tissue (BAT) is the primary site of non-shivering thermogenesis, and activating BAT can effectively reduce obesity and improve associated metabolic diseases 1, 2, 3, 4, 5, 6. Nonshivering thermogenesis in BAT can be activated by environmental and pharmacological stimuli—particularly acute cold exposure, localized heating, and β-adrenergic agonists. By burning stored energy, this process can improve human metabolism and counteract type 2 diabetes, insulin resistance, obesity, and other cardiometabolic diseases 1, 7, 8, 9, 10. In BAT thermogenesis, mitochondrial UCP1 is the primary effector, and its transcription is governed by a complex network of transcription factors, nuclear receptors, and coactivators 11, 12, 13. Recently, Zhao *et al.* (14) demonstrated that ADGRA3 could mitigate diet-induced obesity and enhance glucose homeostasis by promoting UCP1-mediated thermogenesis. Clarifying the regulatory networks that control adipose differentiation and gene expression will therefore improve our understanding of the pathophysiological processes that drive obesity.

In recent years, post-transcriptional RNA modifications have emerged as key regulators of metabolic control and are increasingly recognized as determinants of cellular metabolic states and fate. Among these modifications, N7-methylguanosine (m^7^G) marks are widespread across RNA species and influence gene expression by altering RNA structure, stability, processing, and translation efficiency (15). In mammals, the abundance of m^7^G exhibits considerable heterogeneity across different RNA classes. Its proportion in mRNA is relatively low, ranging from 0.002% to 0.05%, whereas it can constitute 60% to 85% in tRNA. METTL1, as the key m^7^G methyltransferase, forms a complex with the WDR4 protein and cooperatively catalyzes the formation of m^7^G modifications in tRNA (16). As a “writer” protein, METTL1 transfers methyl groups onto various RNA species such as tRNA 17, 18 and miRNA 19, 20 through the coordinated action of its N-terminal S-adenosylmethionine-binding domain and C-terminal RNA recognition domain, thereby influencing multiple dimensions of cellular biology, including immune microenvironment remodeling, regulation of autophagy, modulation of chemosensitivity, and responses to immunotherapy (21). Studies show that elevated METTL1 expression promotes tumor progression by increasing specific tRNA abundance and m^7^G methylation levels (22). Conversely, METTL1 loss activates the rapid tRNA decay (RTD) pathway, leading to widespread clearance of tRNAs bearing m^7^G modifications (23). At the biological functional level, m^7^G-modified tRNAs can selectively modulate the translation of specific mRNA transcripts 24, 25. Dysregulation of tRNA modifications has been widely implicated in tumorigenesis and aging-related pathologies; however, their precise regulatory role in adipose tissue energy metabolism and thermogenic activation remains inadequately explored.

Fluctuations in tRNA stability or structural alterations often trigger specific endonucleolytic cleavages that produce a class of regulatory small RNAs known as tRNA-derived fragments (tRFs/tsRNAs). Recent work shows that tRF expression is highly tissue-specific and temporally dynamic, properties that make these fragments promising biomarkers for disease diagnosis and physiological-state assessment. Accumulating evidence indicates that tRFs modulate gene expression, translation, and epigenetic states by interacting with messenger RNAs (mRNAs) and proteins 26, 27. These features render them promising candidates both for regulating diverse biological processes and as potential biomarkers. Accordingly, we hypothesize that the METTL1-m^7^G modification axis, by governing tRNA stability and the abundance of derived tRFs, may serve as an indispensable hub in thermogenic adipose function and metabolic reprogramming.

This study used cold exposure to induce a classical model of adipose thermogenic activation and applied untargeted metabolomics to profile differential metabolite expression profiles during thermogenic activation. We then validated differences in m^7^G modification across adipose tissue types and linked METTL1-mediated m^7^G modification to adipose thermogenesis. We also quantified the differential distribution of the m^7^G methyltransferase METTL1 in adipose tissues. *In vitro*, we used forskolin to construct a thermogenic activation model in brown adipocytes and identified differential m^7^G modification at the cellular level. Additionally, using TRAC-seq and tRF & tsRNA-seq, we profiled dynamic changes in m^7^G-modified tRNAs and their derived tRFs in BAT during thermogenic activation. From these data, we identified a key regulator of the adipose thermogenic program, tRF-Lys-CTT, which binds the 3′UTR of *Uqcr10* mRNA. RNA immunoprecipitation showed that tRF-Lys-CTT associates with AGO2 to form an RISC complex that targets and silences *Uqcr10*. Because Uqcr10 is an essential structural subunit of mitochondrial electron transport chain complex III, its loss disrupts mitochondrial function. Together, *in vivo* and *in vitro* results link m^7^G modification to adipose thermogenesis and indicate that tRF-Lys-CTT, derived from m^7^G-modified tRNA, limits thermogenic activation by posttranscriptionally repressing *Uqcr10* mRNA.

## Results

### Upregulation of METTL1-mediated m^7^G modification levels during thermogenic activation

m^7^G modification is regulated by the METTL1/WDR4 complex. To investigate the role of m^7^G modification in thermogenic activation of adipose tissue, we first quantified METTL1 and WDR4 expression across different adipose depots. RT-qPCR analysis revealed that *METTL1* mRNA levels were significantly higher in BAT than in white adipose tissues (eWAT and iWAT) (Fig. 1*A*). Western blot and dot blot quantification further confirmed that METTL1 and RNA m^7^G modification levels were higher in BAT compared with WAT (Fig. 1, *B* and *C*). BAT exhibits non-shivering thermogenesis in response to cold stimulation. To establish a thermogenic model of BAT, C57BL/6 mice were housed at room temperature or at 4 °C for 48 h (Fig. 1*D*), with body temperature, food intake, and body weight monitored. On initial exposure to 4 °C, rectal temperature fell sharply and then recovered gradually through endogenous thermogenic mechanisms, stabilizing within 48 h and indicating effective adaptive thermogenesis (Fig. 1*E*). Mice exposed to cold also lost significant body weight (Fig. 1*F*) and, reflecting the higher energy demand for maintaining temperature, consumed substantially more food than room-temperature controls (Fig. 1*G*). Anatomical inspection and weighing showed that cold exposure induced compensatory BAT hyperplasia, as indicated by a significant rise in the BAT weight/body weight ratio (Fig. 1, *H* and *I*). Hematoxylin and eosin (H&E) staining revealed pronounced structural changes in BAT after cold stimulation. Compared with the room-temperature control, adipocytes in the cold-exposed group shifted from large unilocular to smaller multilocular lipid droplets, accompanied by a significant decrease in mean adipocyte area (Fig. 1, *J* and *K*) and a clear increase in cell density (Fig. 1*L*). Transmission electron microscopy (TEM) confirmed that lipid droplets in BAT from the cold-exposed group were more closely packed and smaller than those in the room-temperature group (Fig. 1*M*). Collectively, these findings validate the cold-exposure model and show that cold exposure effectively activates nonshivering thermogenesis in mouse BAT.

**Figure 1:**
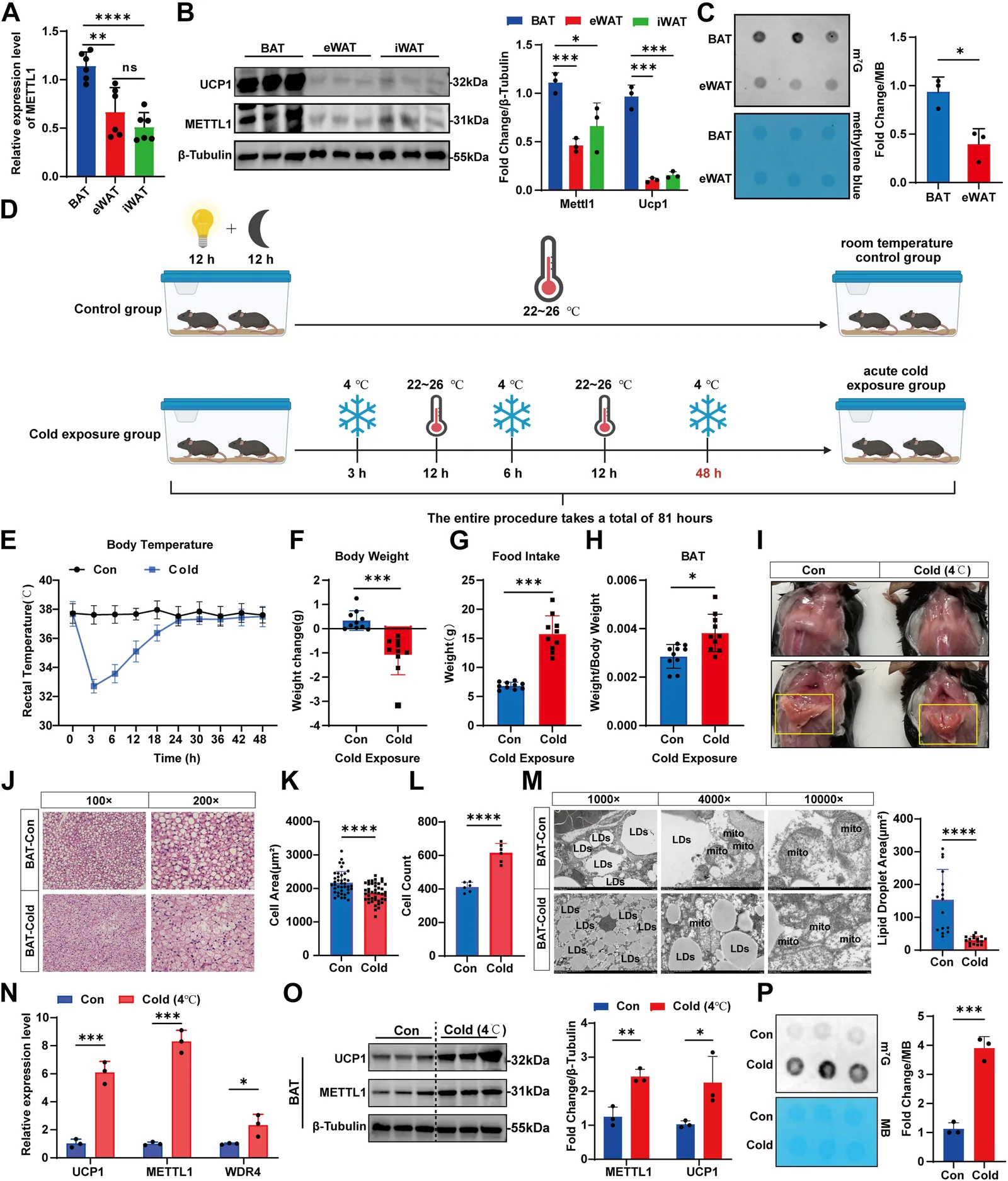
**METTL1-mediated m7G modification levels are upregulated in BAT induced by cold exposure.***A*, RT-qPCR analysis of *METTL1* mRNA expression levels. *B*, Western blot analysis of METTL1 and UCP1 protein expression in multiple adipose tissues with corresponding quantification. *C*, Dot blot analysis of RNA m^7^G modification levels in multiple adipose tissues with corresponding quantification. *D*, Workflow for establishing the cold-exposure mouse model. Created in BioRender. Chen, L. (2027) https://BioRender.com/mxn9vcc. *E*, Rectal temperature measurements of mice (n = 10). *F*, Changes in mouse body weight (n = 10). *G*, Changes in mouse food intake (n = 10). *H*, Ratio of brown scapular adipose tissue weight to body weight. *I*, macroscopic phenotype of mouse scapular brown adipose tissue (BAT). *J-L*, Hematoxylin-eosin (H&E) staining of scapular BAT (*J*), quantification of adipocyte area (*K*), and adipocyte number (*L*). *M*, Transmission electron microscopy (TEM) analysis of the morphology and quantity of lipid droplets and mitochondria in scapular adipose tissue. *N*, changes in mRNA expression levels in BAT from cold-exposed and control mice. *O*, Western blot analysis of UCP1 and METTL1, with densitometric quantification across treatment groups. *P*, measurement and densitometric analysis of RNA m^7^G modification levels (n = 3). Student’s *t* test; data are presented as mean ± SD. ⁎*p* < 0.05, ⁎⁎*p* < 0.01, ⁎⁎⁎*p* < 0.001, ⁎⁎⁎⁎*p* < 0.0001; ns, not significant. Original blot images are provided in the supplementary file 2.

Next, qRT-PCR of BAT after cold exposure showed significant upregulation of *UCP1*, the methyltransferase *METTL1*, and its essential cofactor *WDR4* (Fig. 1*N*). At the protein level, cold exposure substantially increased METTL1 and UCP1 abundance (Fig. 1*O*). Dot blot analysis likewise revealed a pronounced rise in m^7^G modification in mouse BAT after cold exposure, which correlated with adipose thermogenic activation (Fig. 1*P*). Together, these results indicate a close association between the METTL1-m^7^G modification axis and systemic thermogenic activation.

### Metabolic differences during activation of BAT thermogenesis triggered by cold exposure

To further validate the critical role of the m^7^G regulatory system in activating brown adipose tissue thermogenesis from the independent perspective of endogenous small-molecule metabolites, we performed untargeted metabolomic analysis of brown adipose tissue under different treatment conditions. Principal component analysis (PCA) showed clear separation between control (WT-Con) and cold-exposed (WT-Cold) BAT samples in three-dimensional space under both positive and negative ionization modes (Fig. 2, *A* and *B*). To quantify intergroup differences more rigorously, partial least squares discriminant analysis (PLS-DA) produced strong discrimination between BAT from mice kept at room temperature and BAT from mice subjected to cold stimulation (Fig. S1, *A*–*D*), supporting the robustness of the metabolomic results. Volcano plots (Fig. 2, *C* and *D*) showed significant differences in metabolic profiles between the cold-exposed group and the control group under positive and negative ionization modes. In positive ion mode, applying the criteria VIP > 1 and *p* < 0.05, 305 differential metabolites were identified; among these, 322 were significantly upregulated and 139 were significantly downregulated. In negative ion mode, 305 differential metabolites were also identified, of which 218 were significantly upregulated and 87 were significantly downregulated.

**Figure 2:**
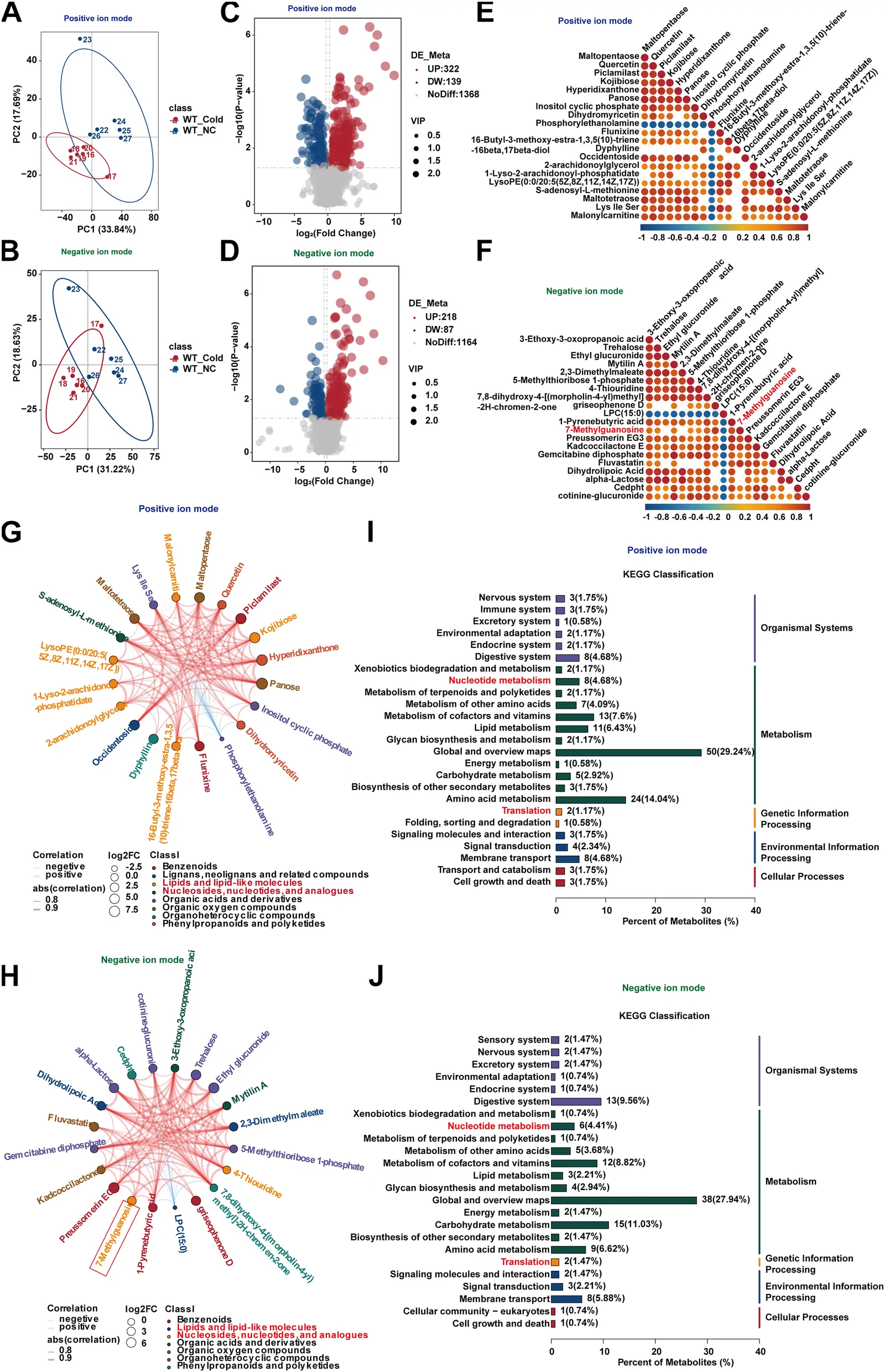
**Metabolic differences during brown adipose tissue thermogenic activation induced by cold exposure.***A-B*, principal component analysis (PCA) score plots under positive ion *A*, and negative ion *B*, modes, comparing room temperature control group (WT-Con, n = 6) and 4 °C cold-exposed group (WT-Cold, n = 6). *C-D*, Volcano plot analysis of differential metabolites (VIP > 1 and *p* < 0.05 considered significant); *red* denotes significantly upregulated metabolites and blue denotes significantly downregulated metabolites. *E-F*, correlation heatmaps of differential metabolites in positive *E* and negative *F* ion modes. *G-H*, Chord diagrams illustrating the correlations and association strengths among differential metabolites in positive *G* and negative *H* ion modes. *I-J*, KEGG enrichment analysis of pathways enriched by differential metabolites in positive (*I*) and negative (*J*) ion modes.

We computed pairwise Pearson correlation coefficients among the differential metabolites to evaluate their interrelationships, and the resulting heatmaps revealed strong collinearity among these metabolites (Fig. 2, *E* and *F*). Notably, several metabolites showed consistent upregulation across groups: in positive-ion mode, S-adenosyl-L-methionine (SAM), maltopentaose, and quercetin; and in negative-ion mode, 7-Methylguanosine and 3-ethoxy-3-oxopropanoic acid (Fig. 2, *E* and *F*). The pronounced enrichment of 7-methylguanosine, a metabolite directly associated with the epitranscriptomic m^7^G modification, therefore confirms that brown adipose tissue undergoes dynamic changes in m^7^G-related metabolites during thermogenic activation (28). Correlation network analysis further indicated that the differential metabolites clustered mainly in lipids and lipid-like molecules, organic acids and derivatives, and nucleosides, nucleotides, and analogues (Fig. 2, *G* and *H*). Notably, 7-Methylguanosine was closely associated with lipids and lipid-like molecules essential for adipose thermogenesis (*e.g.*, LPC(15:0)) as well as with other modified nucleosides (*e.g.*, 4-thiouridine) (Fig. 2*H*). Together, these results indicate that m^7^G modifications may play a potential regulatory role in the activation of adipose tissue thermogenesis.

Subsequently, GSEA enrichment analyses also showed that differential metabolites were enriched in metabolic pathways (Fig. S1, *E* and *F*). KEGG functional classification and pathway enrichment analyses were also performed on the differential metabolites (Fig. 2, *I* and *J*). These analyses showed that cold exposure primarily affected metabolites involved in metabolism-related pathways. In particular, pathways of energy and substrate metabolism (amino acid, carbohydrate, and lipid metabolism) were highly active, supplying substrates and energy for non-shivering thermogenesis in brown adipose tissue. Differential-metabolite enrichment in translation-regulatory pathways indicates an increased cellular demand for protein synthesis under cold stress. Enrichment in nucleotide-related pathways likely reflects dynamic changes in tRNAs and their modifications.

### Brown adipocyte thermogenesis is positively correlated with m^7^G levels

To validate *in vitro* the relationship between adipocyte thermogenesis and m^7^G modification levels, we differentiated C3H10T1/2 cells into brown adipocytes, a standard model for studying brown adipocyte thermogenesis. We used forskolin, an adenylyl cyclase activator that raises intracellular cAMP and activates the cAMP-PKA pathway; by triggering this core thermogenic cascade, forskolin induces lipolysis and thermogenic gene expression and thus functionally mimics cold stimulation. Accordingly, we added forskolin to the differentiation medium of C3H10T1/2 cells undergoing brown adipocyte induction to model cold exposure *in vitro*. Forskolin treatment markedly increased mRNA levels of the thermogenic markers *UCP1*, *PGC-1α*, and the mitochondrial gene *COX8b* (Fig. 3*A*). Protein analysis and immunofluorescence confirmed a corresponding increase in UCP1 protein expression (Fig. 3, *B* and *C*). UCP1 contributes to the regulation of mitochondrial biogenesis in brown adipocytes. We evaluated that Forskolin treatment produced a marked increase in Mito-Tracker fluorescence and a significant rise in mitochondrial DNA (mtDNA) copy number, consistent with enhanced mitochondrial biogenesis (Fig. 3, *D* and *E*). Moreover, Oil Red O (Fig. 3*F*) and BODIPY staining (Fig. 3*G*) showed that Forskolin markedly reduced intracellular lipid droplet size and promoted droplet consumption, indicating a metabolic shift of adipocytes from energy storage to thermogenic energy expenditure. Furthermore, RT-qPCR showed that Forskolin markedly increased mRNA levels of the core m^7^G methyltransferase complex components *METTL1* and *WDR4* (Fig. 3*H*). Dot blot analysis likewise revealed a significant rise in m^7^G modification in adipocytes after thermogenic activation (Fig. 3*I*), suggesting that METTL1-mediated m^7^G modification may contribute critically to thermogenesis regulation. Subsequently, to further validate the effect of METTL1 knockdown on m^7^G modification levels *in vivo*, we generated METTL1 knockout animals using CRISPR/Cas9. Because homozygous knockout (METTL1^−/−^) is embryonically lethal and could not be obtained, we established a stably heritable F1 heterozygous line (METTL1^+/−^). RT-qPCR results showed that *METTL1* expression levels were reduced in BAT of METTL1^+/−^ mice (Fig. 3*Q*), and Dot blot results indicated that METTL1 deficiency downregulated m^7^G modification levels in BAT (Fig. 3*R*).

**Figure 3:**
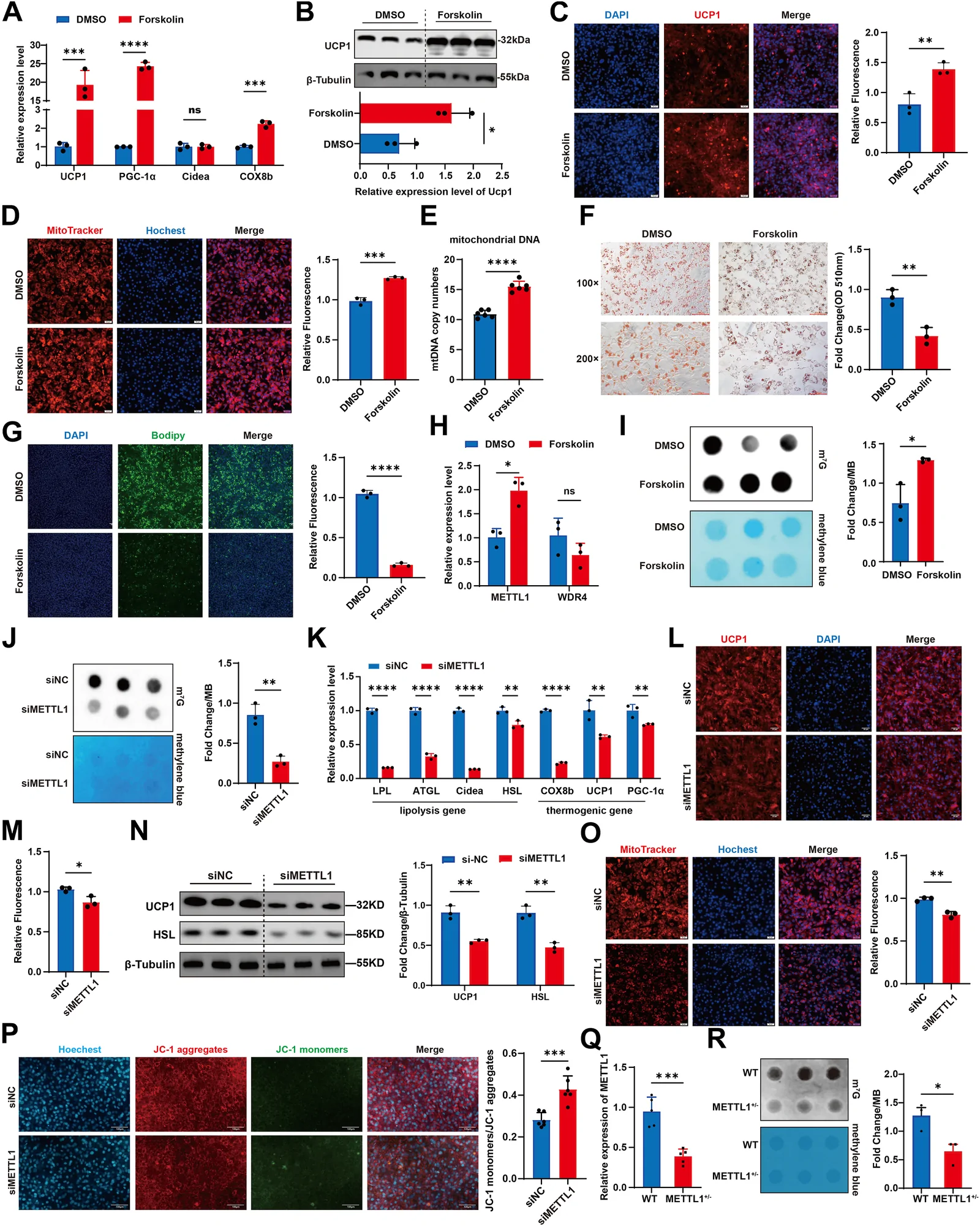
**METTL1-mediated m7G modification participates in the regulation of thermogenic activation of adipocytes.***A*, RT-qPCR analysis of mRNA levels for thermogenic adipocyte markers (*UCP1*, *PGC-1α*, Cidea, *COX8b*) in C3H10T1/2 cells differentiated for 8 days and treated with forskolin for 12 h. *B*, Western blot detection of UCP1 protein in treated cells, with corresponding quantification. *C*,Immunofluorescence staining for UCP1 using an anti-UCP1 primary antibody to assess protein localization and abundance; images and quantification, magnification 200×. *D*, MitoTracker staining to evaluate mitochondrial mass, with fluorescence quantification; magnification 200×. *E*, Measurement of mitochondrial DNA copy number in cells following the respective treatments. *F*, Oil *Red* O staining to assess lipid droplet formation during differentiation, quantified by absorbance at OD 510 nm. *G*, BODIPY fluorescent staining to label intracellular lipid droplets for evaluation of adipogenic differentiation, with fluorescence quantification; magnification 100×. *H*, RT-qPCR analysis of *METTL1* and *WDR4* mRNA expression in cells treated with Forskolin. *I*, dot blot analysis and quantification of RNA m^7^G modification levels in Forskolin-treated cells. *J*, Dot blot analysis and densitometric quantification of m^7^G levels following METTL1 knockdown. *K*, RT-qPCR analysis of mRNA expression for lipolysis-related genes (*LPL*, *ATGL*, *Cidea*, *HSL*) and thermogenesis-related genes (*COX8b*, *UCP1*, *PGC-1α*) after METTL1 knockdown. *L-M*, immunofluorescence staining of UCP1 with quantification of fluorescence intensity (magnification 200×). *N*, Western blot analysis and densitometric quantification of HSL and UCP1 following METTL1 knockdown. *O*, MitoTracker staining to assess mitochondrial content with quantification of fluorescence intensity (magnification 200×). *P*, JC-1 staining to evaluate changes in mitochondrial membrane potential in C3H10T1/2 cells after METTL1 knockdown, with quantification of fluorescence intensity (magnification 200×). *Q*, RT-qPCR analysis of *METTL1* mRNA expression in BAT from WT and METTL1^+/−^ mice. (*R*) Dot blot analysis and quantification of RNA m^7^G modification levels in BAT. n = 3, Student’s *t* test. Data are presented as mean ± SD. ⁎*p* < 0.05, ⁎⁎*p* < 0.01, ⁎⁎⁎*p* < 0.001, ⁎⁎⁎⁎*p* < 0.0001, ns indicates no significant difference.

To investigate the regulatory role of METTL1 in adipose thermogenesis, we knocked down METTL1 by transfecting METTL1 siRNA during differentiation of C3H10T1/2 cells into brown adipocytes. Dot blot analysis showed that METTL1 knockdown significantly reduced RNA m^7^G modification levels (Fig. 3*J*). RT-qPCR demonstrated that siMETTL1 markedly decreased mRNA levels of core thermogenic genes *COX8b*, *UCP1*, and *PGC-1α* (Fig. 3*K*). Immunofluorescence (Fig. 3, *L* and *M*) and Western blotting (Fig. 3*N*) confirmed reduced UCP1 protein after METTL1 knockdown. MitoTracker staining revealed lower mitochondrial fluorescence intensity in the siMETTL1 group (Fig. 3*O*). JC-1 analysis showed an increased fraction of JC-1 monomers (green), a decreased fraction of aggregates (red), and a significantly reduced red/green fluorescence ratio, indicating a loss of mitochondrial membrane potential (Δψm) (Fig. 3*P*). Loss of Δψm impairs respiratory-chain function and thus inhibits fatty acid oxidative catabolism. Consistent with this effect, mRNA expression of lipolysis-related genes *LPL*, *ATGL*, *Cidea*, and *HSL* was markedly suppressed after METTL1 knockdown (Fig. 3*K*).

In addition, we stimulated thermogenic responses with Forskolin while suppressing METTL1 expression. METTL1 knockdown markedly reduced brown adipocyte responses to this stimulation. Immunofluorescent staining of UCP1 showed substantially lower red fluorescence intensity in METTL1-deficient cells than in the Forskolin-treated group (Fig. S2, *A* and *B*). MitoTracker staining likewise indicated that the Forskolin-induced increase in mitochondrial biogenesis was diminished by METTL1 deficiency, as evidenced by reduced mitochondrial fluorescence intensity (Fig. S2, *C* and *D*). Together, these results indicate that METTL1-mediated m^7^G modification is critical for adipose thermogenic activation.

### METTL1-mediated m^7^G modification affects tRNA stability and regulates the generation of thermogenesis-associated tsRNAs in BAT

Numerous studies have shown that m^7^G modifications enhance tRNA stability, and METTL1 knockdown decreases the stability of m^7^G-modified tRNAs. To define m^7^G-modified tRNA expression in adipose tissue, we profiled the m^7^G-modified tRNA repertoire in BAT and eWAT using TRAC-seq. The volcano plot identified differentially expressed m^7^G-modified tRNAs between BAT and eWAT (Fig. 4*A*). Comparing with eWAT, we detected 226 differentially expressed tRNAs, which were then categorized and quantified in BAT (Fig. 4*B*). Environmental stress can trigger precise cleavage of mature tRNAs to produce tsRNAs and tRFs. Reduced m^7^G modification destabilizes tRNAs and increases their susceptibility to cleavage, promoting tsRNA generation. To examine whether tsRNAs derived from m^7^G-modified tRNAs are linked to adipose thermogenesis, we profiled tsRNA expression using tRF & tsRNA-seq. Overall, most tsRNAs commonly expressed in BAT and eWAT originated from mature tRNAs (48.9%), and 57.4% of those mature tRNA-derived tsRNAs came from tRNAs carrying m^7^G modifications (Fig. 4*C*). Among the 166 tsRNAs derived from m^7^G-modified tRNAs, most originated from tRNA species such as GluTTC, GlyCCC, and TyrGTA (Fig. 4*D*). Differential expression analysis of mature tRNA-derived tsRNAs in BAT and eWAT (log2(fold change) ≥ 1.0 and *p* < 0.05) identified sets of significantly altered tsRNAs, which are displayed in a heatmap (Fig. 4*E*). We selected tsRNAs that exhibited significant differential expression and high expression levels for subsequent KEGG enrichment analysis. Notably, the target genes of tRF-1:32-Lys-CTT (tRF-Lys-CTT), which was significantly downregulated in BAT, were enriched in signaling pathways such as Thermogenesis and Oxidative Phosphorylation (Fig. 4*F*). This finding aligns with our previously described metabolomic sequencing results and suggests that tRF-Lys-CTT may be closely linked to the regulation of thermogenesis activation.

**Figure 4:**
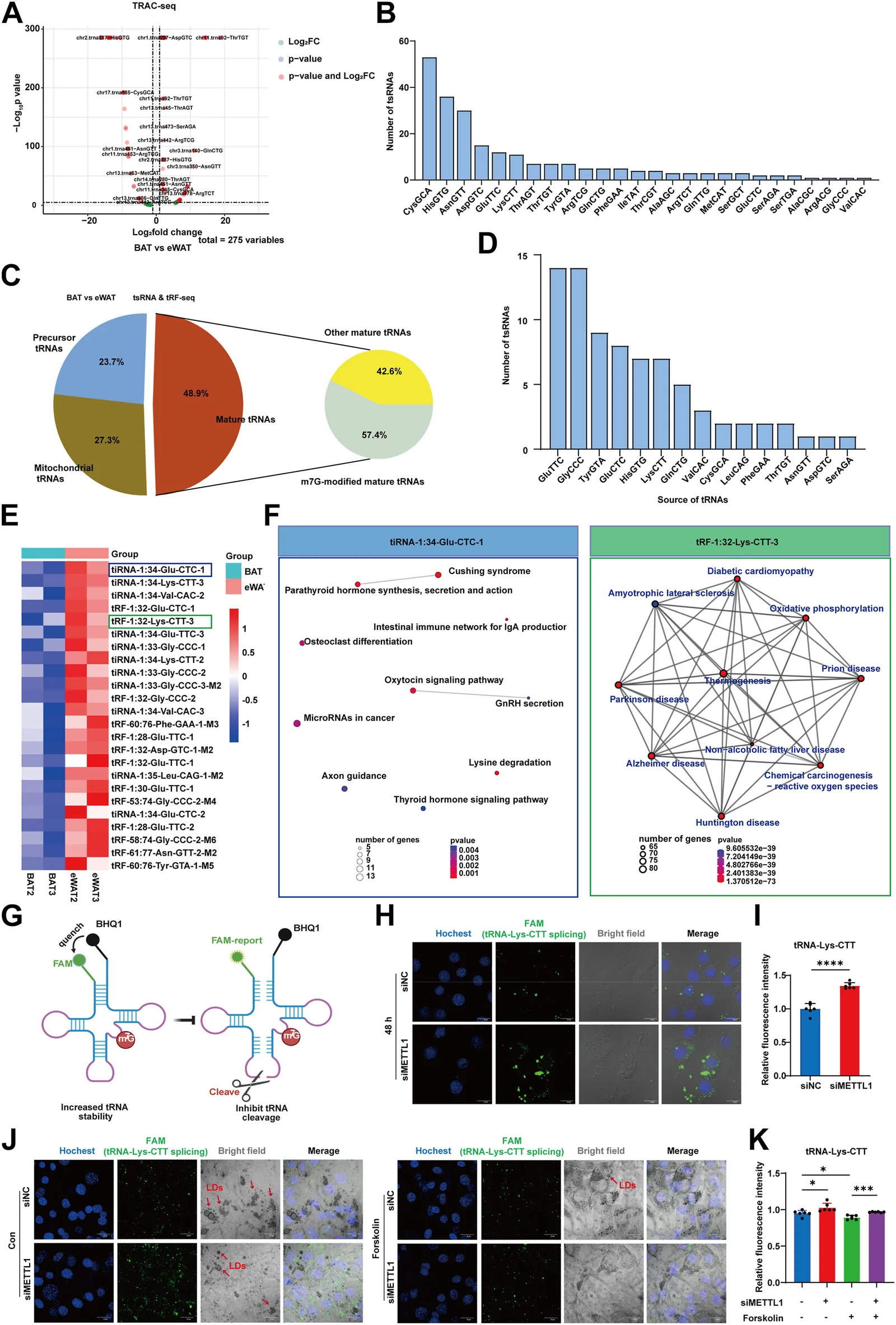
**Differential characterization of m7G-modified tRNAs and their derived tsRNAs during thermogenic activation.***A*, Identification of m^7^G-modified tRNAs by TRAC-seq, with a volcano plot showing differential expression profiles between BAT and eWAT. *B*, Classification statistics for the differentially expressed tRNAs. (*C*-*D*) Quantification of tsRNA species derived from parent tRNAs that carry m^7^G and are commonly expressed in BAT and eWAT. *E*, Heatmap of differentially expressed tsRNAs. *F*, KEGG analysis of regulatory pathways associated with predicted target genes of tRF-1:32-Lys-CTT-3 and tiRNA-1:34-Glu-CTC-1. *G*, Design of a tRNA fluorescence-quenching reporter system (FAM-BHQ1) and a schematic of expected tRNA cleavage patterns. *H*, Knockdown of METTL1 by siMETTL1 in proliferating C3H10T1/2 cells, combined with introduction of the tRNA fluorescence-quenching reporter to monitor cleavage of tRNA-Lys-CTT. *I*, Quantification of relative fluorescence intensity following tRNA cleavage. *J-K*, Monitoring tRNA cleavage with the fluorescence-quenching reporter in C3H10T1/2 cells during differentiation after Forskolin treatment or METTL1 knockdown (*J*) and quantification of relative fluorescence intensity (*K*). Statistical analysis by Student’s *t* test, data presented as mean ± SD. ⁎*p* < 0.05, ⁎⁎⁎⁎*p* < 0.0001, ns, not significant.

Subsequently, we validated the regulation of the parental tRNA from which tRF-Lys-CTT is derived by METTL1-mediated m^7^G modification. We constructed a fluorescence quenching reporter system (FAM-BHQ1) to monitor tRNA cleavage (Fig. 4*G*). A FAM fluorophore was appended to the 5′ end of the tRNA and a BHQ1 dark quencher to the 3′ end; when the probe is intact, FAM excitation energy is absorbed by BHQ1, thereby suppressing the fluorescent signal. We targeted METTL1 knockdown in proliferating C3H10T1/2 cells and observed that, in the siMETTL1 group, the FAM green fluorescence signal of tRNA-Lys-CTT was markedly increased and exhibited prominent punctate aggregation, indicating that in the absence of METTL1-mediated m^7^G modification protection, the tRNA is more susceptible to nuclease-mediated cleavage (Fig. 4*H*). Quantification of fluorescence intensity also showed that the relative fluorescence in the siMETTL1 group was significantly elevated compared with the control group (Fig. 4*I*). Similarly, in mature adipocytes (with clearly visible lipid droplets, LDs), METTL1 knockdown exhibited significantly higher FITC signal, confirming that METTL1 loss induces fragmentation of tRNA-Lys-CTT and generation of tRFs. Under Forskolin-induced thermogenic activation, intracellular levels of tRNA cleavage products showed a downward trend. Moreover, upon Forskolin stimulation, METTL1 knockdown markedly rescued the Forskolin-induced reduction in fluorescence signal, restoring tRNA cleavage levels. Relative quantification of fluorescence intensity also revealed statistically significant differences (Fig. 4, *J* and *K*). These results demonstrate that METTL1, by installing m^7^G modifications on tRNAs, preserves the stability of tRNA-Lys-CTT during adipocyte thermogenic activation.

### tRF-Lys-CTT inhibits adipocyte differentiation and thermogenic activation

We investigated whether tRF-Lys-CTT, a tRNA fragment derived from an m^7^G-modified tRNA, affects cellular thermogenic activation. Firstly, we compared tsRNA expression across adipose tissue. Consistent with the tsRNA-seq data, tsRNAs originating from m^7^G -modified tRNAs (tRF-1:34-Glu-CTC-1, tRF-1:32-Lys-CTT-3, and tRF-60:76-Tyr-GTA-1-M5) were substantially more abundant in eWAT and iWAT than in BAT (Fig. 5*A*). In adipocytes, Forskolin-induced thermogenic stimulation reduced the levels of these tsRNAs, simultaneous knockdown of METTL1 prevented this Forskolin-induced suppression (Fig. 5*B*). Similarly, downregulated expression of tRF-Lys-CTT was detected in BAT treated with cold stimulation (Fig. 5*C*). To investigate the role of tRF-Lys-CTT in brown adipocyte thermogenesis, we overexpressed it during differentiation of C3H10T1/2 cells (Fig. 5*D*). Gain-of-function analysis revealed that elevated tRF-Lys-CTT significantly reduced mRNA levels of core thermogenic and lipolysis-related genes (Fig. 5*E*). Western blotting confirmed corresponding decreases at the protein level (Fig. 5*F*). Immunofluorescence further showed that tRF-Lys-CTT overexpression markedly suppressed UCP1 protein expression (Fig. 5*G*). Furthermore, Mito-tracker staining showed that tRF-Lys-CTT overexpression reduced mitochondrial fluorescence intensity (Fig. 5*H*). The JC-1 assay revealed that high tRF-Lys-CTT levels increased the fraction of JC-1 monomers, indicating a loss of mitochondrial membrane potential (ΔΨm) (Fig. 5*I*). Bodipy staining further showed fewer intracellular lipid droplets in cells overexpressing tRF-Lys-CTT, consistent with impaired adipogenic differentiation (Fig. 5*J*). Together, these findings indicate that aberrant upregulation of tRF-Lys-CTT compromises mitochondrial function and thermogenic metabolic capacity.

**Figure 5:**
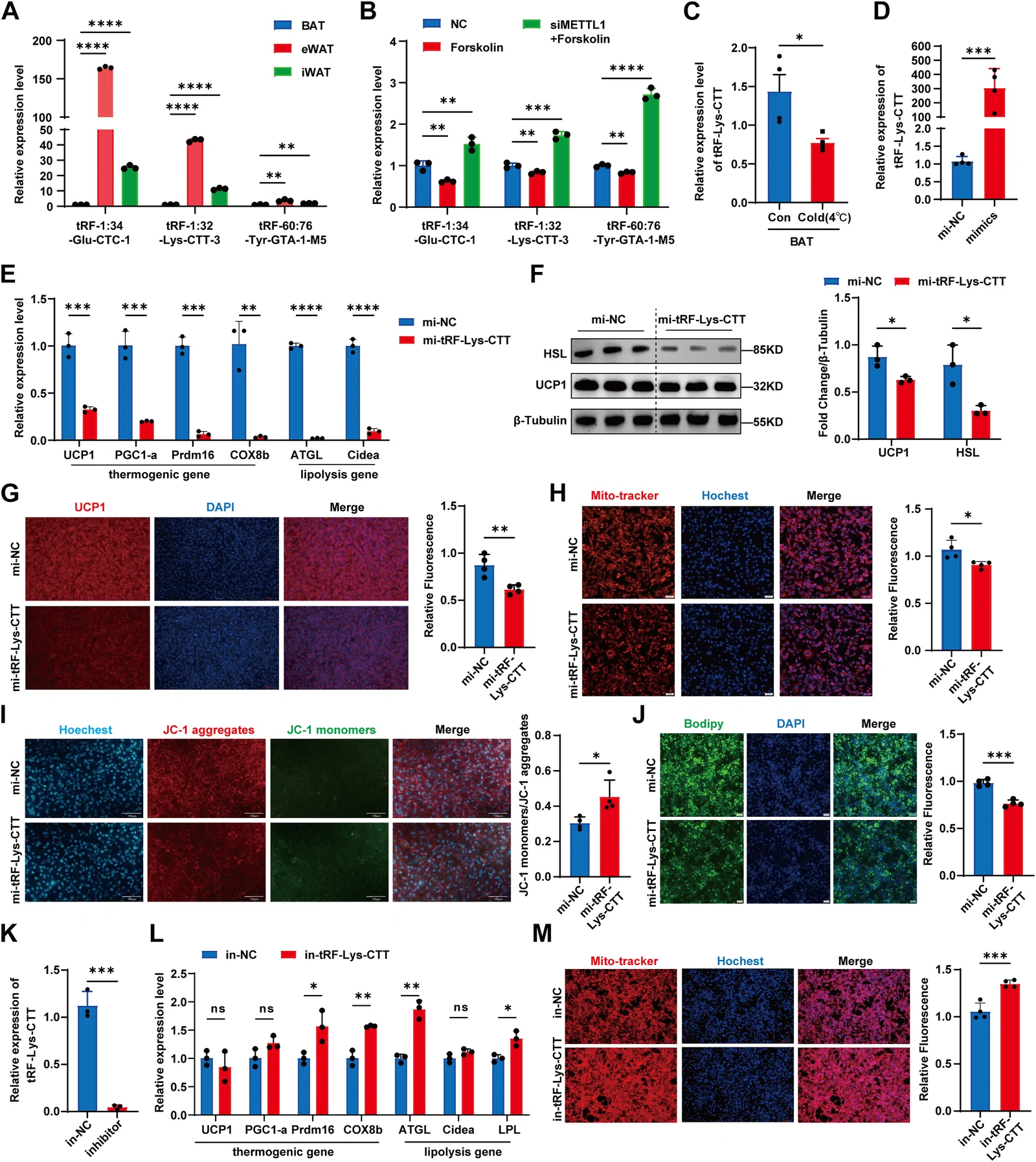
**tRF-Lys-CTT inhibits adipocyte differentiation and thermogenic activation.***A*, RT-qPCR analysis of tsRNA expression (tRF-1:34-Glu-CTC-1, tRF-1:32-Lys-CTT-3, tRF-60:76-Tyr-GTA-1-M5) across distinct adipose depots. *B*, RT-qPCR analysis of tsRNA levels in C3H10T1/2 cells treated with forskolin and in which METTL1 was knocked down. *C*, RT-qPCR analysis of tRF-1:32-Lys-CTT-3 (tRF-Lys-CTT) expression in BAT from cold-exposed and control mice. *D*, RT-qPCR quantification of transfection efficiency in C3H10T1/2 cells 8 days after transfection with tRF-Lys-CTT mimics (mi-tRF-Lys-CTT). *E*, RT-qPCR quantification of mRNA expression for thermogenic genes (*UCP1*, *PGC-1α*, *Prdm16*, *COX8b*) and lipolytic genes (*ATGL*, *Cidea*). *F*, Western blot analysis of HSL and UCP1 protein levels with densitometric quantification of immunoblots. *G*, Immunofluorescence staining using an anti-UCP1 antibody with fluorescence intensity quantification; magnification, 100×. *H*, MitoTracker staining of mitochondria with fluorescence intensity quantification; magnification, 100×. (*I*) JC-1 staining to assess mitochondrial membrane potential with quantitative analysis; magnification, 200×. *J*, BODIPY staining of lipid droplets in differentiated cells with fluorescence intensity quantification; magnification, 100×. *K*, RT-qPCR quantification of transfection efficiency 8 days after transfection with tRF-Lys-CTT inhibitors (in-tRF-Lys-CTT). *L*, RT-qPCR quantification of mRNA expression for thermogenic genes (*UCP1*, *PGC-1α*, *Prdm16*, *COX8b*) and lipolytic genes (*ATGL*, *Cidea*, *LPL*). *M*, MitoTracker staining of mitochondria with fluorescence intensity quantification after transfection with tRF-Lys-CTT inhibitors; magnification, 100×. n = 3. Student’s *t* test. Data are presented as mean ± SD. ⁎*p* < 0.05, ⁎⁎*p* < 0.01, ⁎⁎⁎*p* < 0.001, ⁎⁎⁎⁎*p* < 0.0001; ns, not significant.

Subsequently, inhibition of tRF-Lys-CTT expression in cells produced the opposite phenotype (Fig. 5*K*). RT-qPCR analysis showed that suppressing tRF-Lys-CTT significantly increased mRNA levels of the thermogenesis-associated genes *Prdm16* and *COX8b*, as well as the lipolysis-related genes *ATGL* and *LPL* (Fig. 5*L*). Cells with reduced tRF-Lys-CTT also exhibited greater mitochondrial content (Fig. 5*M*), indicating induced thermogenic activation. Together, these findings demonstrate that tRF-Lys-CTT modulates adipocyte thermogenic activation and acts as an inhibitor of the cellular thermogenic program.

### Differential profiling of protein translation during BAT thermogenesis activation

Previous studies have shown that METTL1 can modulate mRNA translation by catalyzing m^7^G modification on tRNA molecules (Metabolic rewiring during bone development underlies tRNA m^7^G-associated primordial dwarfism). To clarify how METTL1-mediated m^7^G modification influences adipose thermogenesis, we performed Ribo-seq and RNA-seq to profile the translatome and transcriptome of murine brown adipose tissue (BAT) at room temperature and after cold exposure. Transcriptome sequencing indicated that cold stimulation substantially altered the transcriptional steady state of BAT. Hierarchical clustering of the expression data revealed that, relative to controls, 823 genes were downregulated and 1185 genes were upregulated at the transcript level in BAT after cold exposure (Fig. S3*A*). KEGG and GO enrichment analyses showed that the downregulated genes (Down DEGs) clustered mainly in structural pathways, including extracellular matrix (ECM)-receptor interaction and cell adhesion molecules (Fig. S3, *B* and *D*). By contrast, the upregulated DEGs were enriched in metabolic and functional modules such as Peroxisome, Glycerophospholipid metabolism, PPAR signaling pathway, and fatty acid metabolism (Fig. S3, *C* and *E*). Together, these results indicate that cold exposure shifts BAT from a transcriptional program that maintains tissue structural homeostasis toward an active thermogenic metabolic program.

To examine changes in protein synthesis, we performed ribosome profiling (Ribo-seq), which revealed translational reprogramming under cold stress. A volcano plot of differentially expressed proteins (DEPs) in BAT after cold exposure identified 93 downregulated and 145 upregulated DEPs (Fig. 6*A*). Proteins with reduced translation (Down DEPs) were enriched in pathways associated with the ribosome, ribosome biogenesis, and rhythm regulation (Fig. 6, *B* and *D*). In contrast, KEGG analysis showed that genes with increased translation (Up DEPs) were significantly enriched in metabolic pathways critical for thermogenesis, including the PPAR signaling pathway, fatty acid metabolism, glycerophospholipid metabolism, and glycolysis/gluconeogenesis (Fig. 6*C*). GO enrichment further confirmed strong activation of cofactor metabolism, coenzyme metabolism, and redox processes (Fig. 6*E*). These results align closely with the transcriptomic data, support the maintained abundance of UCP1 and other thermogenesis-related genes, and indicate that cold exposure enhances thermogenic metabolism by increasing translation of enzymes involved in heat production. The integrated Ribo-seq and RNA-seq scatterplot (Fig. 6*F*) showed that the majority of thermogenesis-related genes exhibit homodirectional changes, indicating coordinated up- or downregulation at both the transcriptional and translational levels. A substantial subset, however, changes significantly only at the translational level (OnlyRibo), which highlights an independent role for post-transcriptional regulation in the thermogenic stress response. Calculation of translation efficiency (TE) further shows that cold stimulation provokes widespread translational remodeling in BAT (Fig. 6*G*). Analysis of molecular functions (GO MF) and interaction networks revealed that genes exhibiting differential TE were predominantly enriched in GTP binding, guanyl nucleotide binding, RNA splicing, and ribosome biogenesis (Fig. 6, *H* and *I*). Together, these findings indicate that BAT adapts to cold-induced thermogenic activation through both transcriptional control and selective modulation of translation efficiency that reprograms metabolic pathways.

**Figure 6:**
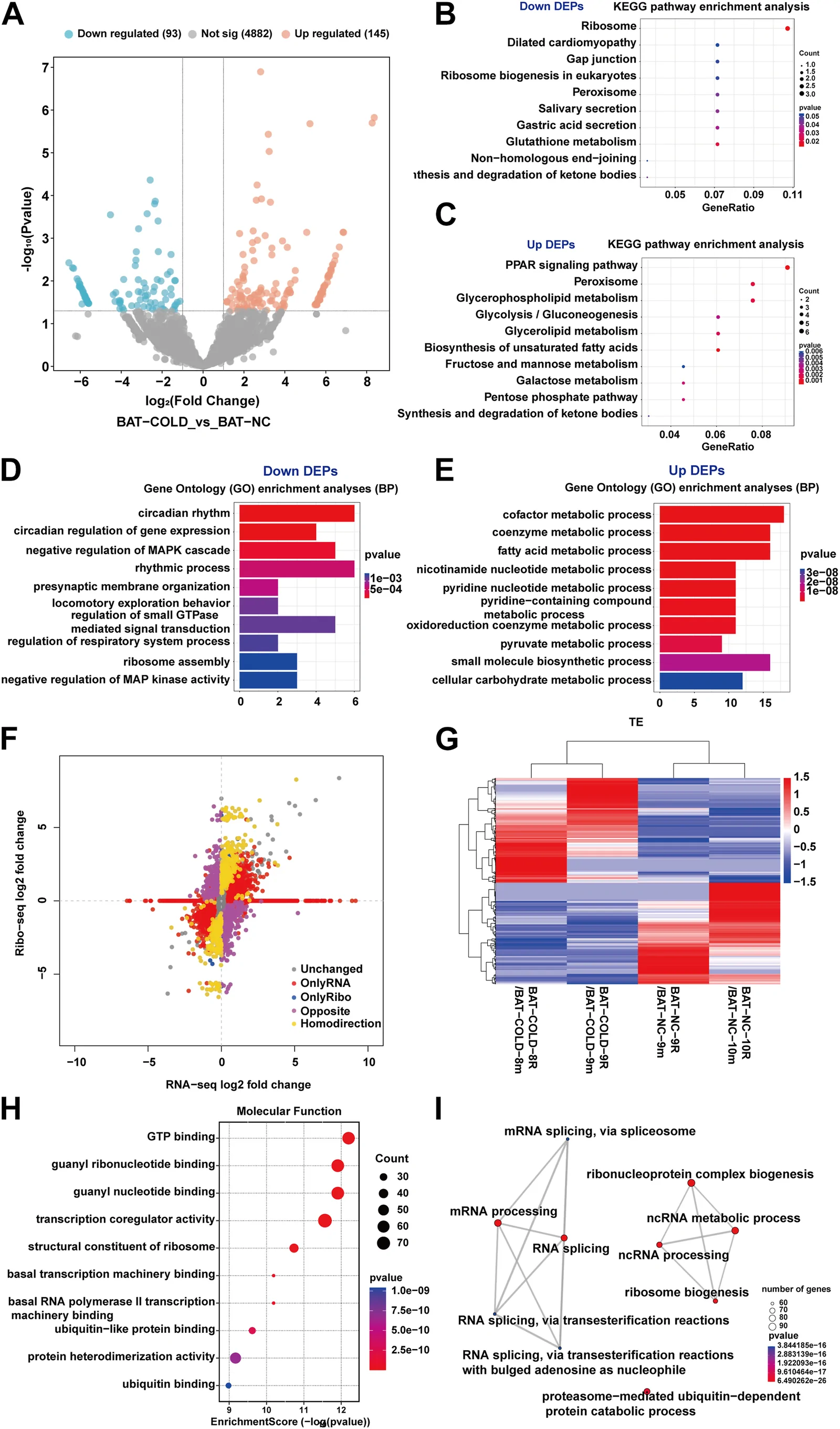
**Ribo-seq analysis of genes during BAT thermogenic activation.***A*, Volcano plot of differentially expressed proteins (DEPs) identified by Ribo-seq (|log2FC| > 1, *p* < 0.05). *B-C*, KEGG pathway enrichment analysis of DEPs. *D-E*, GO enrichment analysis of DEPs. *F*, Four-quadrant plot showing translational regulation by integrating Ribo-seq and RNA-seq data. *G*, Creating a Heat Map of Translation Efficiency. *H-I*, GO enrichment analysis of genes with altered translation efficiency, Molecular Function (*H*), Biological Process (*I*).

### tRF-Lys-CTT modulates thermogenic activation in adipocytes by targeting *Uqcr10*

To test whether METTL1 affects global translation, we knocked down METTL1 and measured puromycin incorporation to quantify nascent peptide synthesis. Western blotting showed that METTL1 inhibition reduced puromycin labeling of proteins, indicating a decline in overall translation (Fig. 7*A*). Consistently, transfection with a tRF-Lys-CTT mimic (mi-tRF-Lys-CTT) also lowered the overall abundance of puromycin-labeled bands (Fig. 7*A*). To further molecular targets through which tRF-Lys-CTT modulates adipose thermogenesis, we used four bioinformatic tools (tRFTar, TargetScan, miRDB, tRFtarget 2.0) to predict its potential downstream genes. We intersected these predicted targets with differentially expressed genes using a Venn diagram (Fig. S4*A*) and obtained three thermogenesis-related candidates: *Uqcr10*, *Slc25a20*, and *Acsl5*. RT-qPCR confirmed that all three genes were significantly upregulated in mouse BAT after cold exposure (Fig. S4*B*. Forskolin treatment similarly increased their expression in C3H10T1/2 cells (Fig. S4*C*). Importantly, METTL1 knockdown substantially reduced the Forskolin-induced upregulation of *Uqcr10*, indicating that Uqcr10 is a likely downstream effector of the METTL1/tRF-Lys-CTT axis. To further clarify the targeting relationship between tRF-Lys-CTT and *Uqcr10*, we overexpressed or knocked down tRF-Lys-CTT in C3H10T1/2 cells. RT-qPCR analysis showed that *Uqcr10* mRNA levels were significantly decreased following tRF-Lys-CTT overexpression, whereas inhibition of tRF-Lys-CTT resulted in increased *Uqcr10* expression (Fig. 7*B*). Sequence analysis predicted that tRF-Lys-CTT can bind to the 3′UTR of *Uqcr10* through complementary base pairing (Fig. S4*D*). A dual-luciferase reporter assay validated this interaction: tRF-Lys-CTT markedly reduced luciferase activity of the reporter carrying the *Uqcr10*-WT 3′UTR, and this repression was lost when the predicted binding site was mutated (MUT) (Fig. 7*C*).

**Figure 7:**
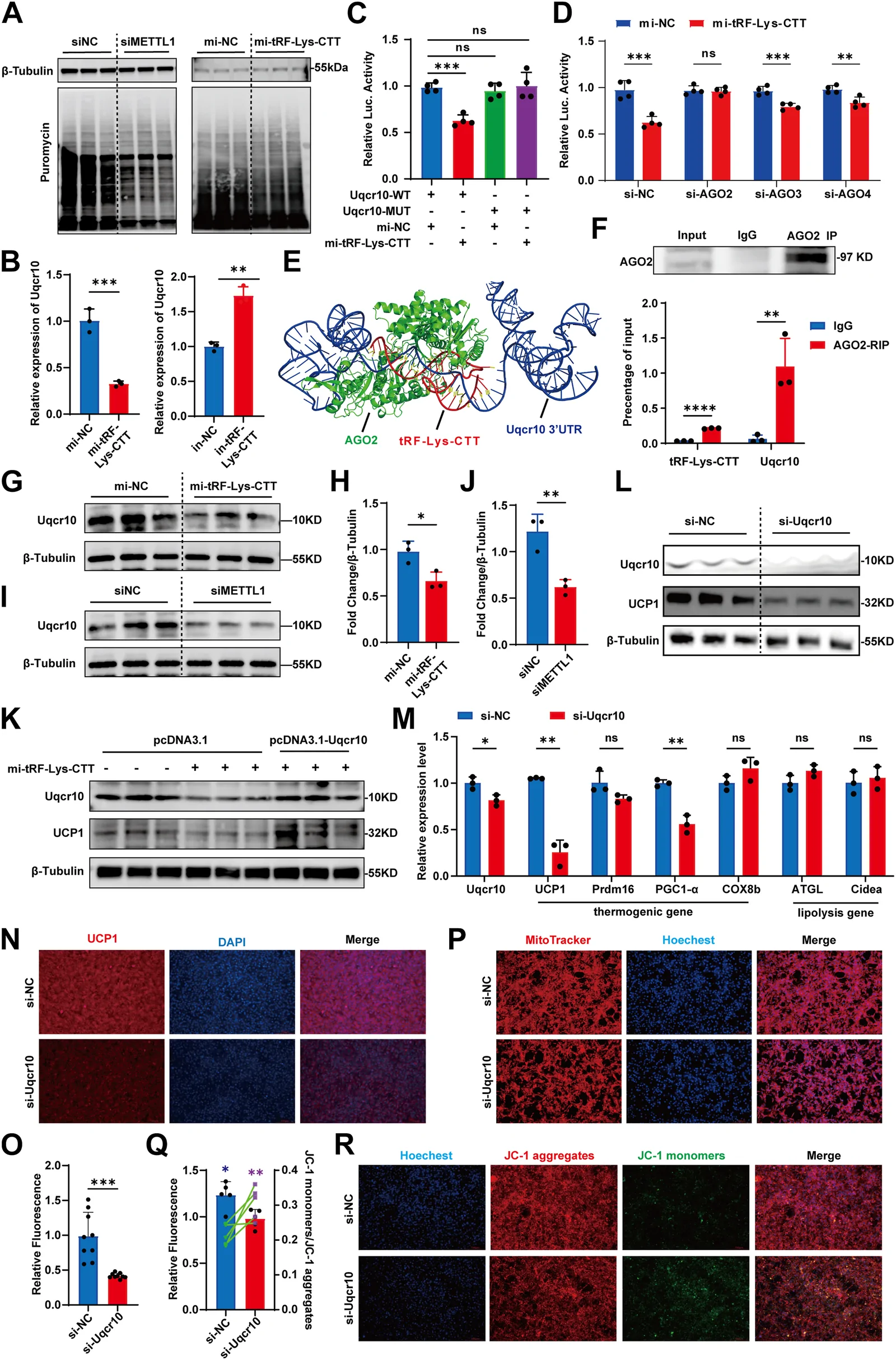
**tRF-Lys-CTT regulates thermogenic activation of adipocytes by targeting Uqcr10.***A*, puromycin incorporation assays assessed protein synthesis after METTL1 knockdown (*Left*) or tRF-Lys-CTT overexpression (*Right*); β-tubulin served as the loading control for each group. *B*, RT-qPCR quantification of *Uqcr10* mRNA in C3H10T1/2 cells after transfection with tRF-Lys-CTT mimics or inhibitors. *C*, Synthesis of wild-type (WT) and mutant (MUT) *Uqcr10* 3′UTR constructs and validation of their interaction with tRF-Lys-CTT using a dual-luciferase reporter assay. *D*, evaluation of the *Uqcr10*–tRF-Lys-CTT interaction by dual-luciferase assay following Argonaute knockdown (si-NC, si-AGO2, si-AGO3, si-AGO4). *E*, schematic 3D molecular-docking model of AGO2 (*green*), tRF-Lys-CTT (*red*), and the *Uqcr10* 3′UTR (*blue*) predicted by AlphaFold3 (https://alphafoldserver.com/). *F*, RIP-qPCR detection of AGO2, *Uqcr10*, and tRF-Lys-CTT binding; Western blot confirms high immunoprecipitation efficiency and specificity of the AGO2 antibody. (*G*-*H*) Western blot and densitometric analysis of Uqcr10 in cells transfected with tRF-Lys-CTT mimics. *I-J*, Western blot and densitometric analysis of Uqcr10 in cells transfected with siMETTL1. (*K*) Western blot analysis of Uqcr10 and UCP1 after co-transfection with pcDNA3.1-Uqcr10 and tRF-Lys-CTT mimics. *L*, Western blot analysis of Uqcr10 and UCP1 after Uqcr10 knockdown. *M*, RT-qPCR analysis of thermogenic genes (*UCP1*, *Prdm16*, *PGC-1α*, *COX8b*) and lipolysis-related genes (*ATGL*, *Cidea*) following si-Uqcr10 transfection. *N-O*, immunofluorescent staining of UCP1 across treatment groups (*N*) with fluorescence intensity quantification (*O*); magnification, 100×. *P-Q*, MitoTracker staining of mitochondria with subsequent fluorescence intensity quantification; magnification, 100×. *R*, JC-1 staining to assess mitochondrial membrane potential with fluorescence intensity quantification; magnification, 100×. n = 3. Student’s *t* test. Data are presented as mean ± SD. ⁎*p* < 0.05, ⁎⁎*p* < 0.01, ⁎⁎⁎*p* < 0.001; ns, not significant.

Because some tsRNAs can act like microRNAs by forming complexes with Argonaute (AGO) proteins to silence genes, we tested which AGO is required for tRF-Lys-CTT function. With tRF-Lys-CTT overexpressed, we knocked down AGO2, AGO3, and AGO4 individually using specific siRNAs. Only AGO2 depletion fully restored the luciferase activity suppressed by tRF-Lys-CTT; knockdown of AGO3 or AGO4 did not reverse the repression (Fig. 7*D*). These results indicate that AGO2 is likely the indispensable catalytic component of tRF-Lys-CTT–mediated gene silencing. Subsequently, we constructed a three-dimensional structural model illustrating the interaction mechanism among AGO2 protein, tRF-Lys-CTT, and *Uqcr10* 3′UTR (Fig. 7*E*). To confirm physical assembly of this silencing complex, we performed RIP-qPCR with an anti-AGO2 antibody. Compared with controls, AGO2 was enriched for endogenous tRF-Lys-CTT and *Uqcr10* mRNA (Fig. 7*F*). These results indicate that tRF-Lys-CTT associates with AGO2 to form a functional RISC that directly represses Uqcr10 posttranscriptionally. Consistent with this mechanism, cells overexpressing tRF-Lys-CTT showed a pronounced reduction in Uqcr10 protein (Fig. 7, *G* and *H*). Conversely, METTL1 knockdown similarly decreased Uqcr10 protein levels (Fig. 7, *I* and *J*). To test whether Uqcr10 is the downstream effector of tRF-1:32-Lys-CTT-3, we performed rescue experiments. Brown adipocytes transfected with a tRF-1:32-Lys-CTT-3 mimic (mi-tRF-Lys-CTT) were co-transfected with an Uqcr10 overexpression construct. As expected, mi-tRF-Lys-CTT alone markedly reduced endogenous Uqcr10 protein levels, and the thermogenic core protein UCP1 decreased correspondingly. Co-overexpression of Uqcr10, however, restored Uqcr10 protein levels and reversed the post-transcriptional silencing induced by mi-tRF-Lys-CTT. The suppression of UCP1 by mi-tRF-Lys-CTT was likewise abolished (Figs. 7*K* and S4*E*). Together, these data indicate that tRF-Lys-CTT impairs mitochondrial biogenesis and thermogenic function in brown adipocytes by post-transcriptionally repressing Uqcr10.

Furthermore, we generated a Uqcr10-deficient model during C3H10T1/2 cell differentiation by transfecting cells with si-Uqcr10. RT-qPCR showed that Uqcr10 downregulation directly reduced mRNA levels of the thermogenic regulator *PGC-1α* and the effector *UCP1*, while lipolysis-related genes *ATGL* and *Cidea* remained unchanged (Fig. 7*M*). Western blotting (Figs. 7*L* and S4*F*) and immunofluorescence (Fig. 7, *N* and *O*) both showed decreased UCP1 after Uqcr10 knockdown. Because Uqcr10 is an essential subunit of mitochondrial electron transport chain complex III, we assessed mitochondrial function with MitoTracker and JC-1 staining. Uqcr10 knockdown reduced MitoTracker fluorescence (Fig. 7, *P* and *Q*) and significantly increased the JC-1 monomer/aggregate ratio, indicating mitochondrial membrane depolarization (Fig. 7, *Q* and *R*). Together, these results indicate that Uqcr10 is required to sustain thermogenic activation in BAT.

## Discussion

Thermogenic plasticity of adipose tissue is a primary defense against cold exposure and a mechanism for disposing of excess energy. Prior research has focused mainly on transcription factor networks (*e.g.*, PRDM16, PPARγ, PGC-1α) and on epigenetic processes that initiate and maintain thermogenic gene programs. In contrast, this study examines noncoding RNA–mediated translational control and identifies a key role for tRNA-derived fragments in reprogramming lipid metabolism. We find that thermogenic activation involves more than transcriptional upregulation; it also entails pronounced remodeling of tRNA stability and cleavage dynamics. This stress-induced change in tRNA metabolism creates flexible regulatory nodes that let cells rapidly adjust protein synthesis and thereby compensate for the slower timescale of canonical transcriptional controls. tRNAs are essential regulators of genetic-code decoding and intracellular protein synthesis, and they play central roles in many biological processes 29, 30. Numerous chemical modifications decorate tRNAs and are critical to their function. In particular, modifications within the anticodon region are indispensable for accurate translation and for supporting organismal growth 31, 32.

This study employed untargeted metabolomics to delineate the metabolic landscape of brown adipose tissue (BAT) upon cold exposure. Notably, S-adenosyl-L-methionine (SAM), a principal methyl donor, was markedly upregulated in thermogenically activated BAT. The elevation of SAM not only reflects an accelerated methionine cycle during thermogenesis but also metabolically supports increased demand for RNA modifications. Among these, m^7^G represents an evolutionarily highly conserved RNA modification. The methyltransferase METTL1 forms a stable complex with its cofactor WDR4 and, *via* its αC and α6 helical domains, specifically binds the tRNA variable loop to catalyze N7-methylation of guanosine at position 46, thereby enhancing tRNA conformational stability 33, 34. At the post-transcriptional level, tRNA m^7^G modification catalyzed by the METTL1-WDR4 complex regulates translational efficiency. METTL1 supplies the methyltransferase activity and directly catalyzes the methylation, while WDR4 acts as a scaffold that preserves the complex’s structural integrity and function (25). Recent studies show that aberrant METTL1-dependent tRNA m^7^G modification increases the translational efficiency of multiple oncogenes and thereby promotes proliferation, migration, and invasion of hepatocellular carcinoma cells, indicating that this modification axis is a key driver of tumor initiation and progression (35). However, the specific roles and molecular mechanisms by which METTL1-mediated tRNA m^7^G modifications influence adipose thermogenesis remain unclear. In this study, we systematically map the dynamic changes in tRNA modifications and dissect the metabolic regulatory mechanism of the derived fragment tRF-Lys-CTT during activation of thermogenic adipocytes. We show that cold exposure substantially remodels tRNA modification profiles in brown adipose tissue and that the abundance of specific tRNAs responds sensitively to temperature changes.

tsRNAs/tRFs are a class of novel small noncoding RNAs derived from tRNAs, whose biogenesis primarily depends on specific nucleases cleaving precursor or mature tRNAs. tsRNAs/tRFs play key roles in cellular processes such as epigenetic modification, maintenance of mRNA stability, and regulation of translation 36, 37. For example, 5′-tiRNA-His-GTG promotes colorectal cancer progression by downregulating translation of *LATS2* mRNA (37). In addition, tRF-21 is regulated by inflammatory factors and hnRNP L and suppresses the progression of pancreatic cholangiocarcinoma (38). Recent studies show that modified tRFs are closely linked to the pathogenesis of multiple diseases. For example, endogenous tRFs carrying m^5^C and m^2^G modifications in sperm from male mice fed a high-fat diet induce metabolic disturbances in offspring, whereas unmodified synthetic tRFs do not produce similar effects, indicating that RNA modifications determine tRF functional activity (39).

In this study, we identified differentially expressed tRFs generated by tRNA cleavage following m^7^G modification during the activation of adipose thermogenesis and subsequently performed functional validation. Under thermogenic activation, METTL1-mediated m^7^G modification effectively suppressed tRNA cleavage, thereby reducing the accumulation of tRF-Lys-CTT. Mechanistically, we show that tRF-Lys-CTT is a key regulator of adipose thermogenesis and that it acts by targeting Uqcr10 to modulate the mitochondrial respiratory chain. Uqcr10 is a critical subunit of mitochondrial electron transport chain complex III and plays a key role in oxidative phosphorylation across the inner mitochondrial membrane (40). Downregulation of ATP5G1, UQCR10, and NDUFA3 has been shown to reduce ATP production, and the resulting decline in ATP synthesis is often accompanied by increased mitochondrial reactive oxygen species (ROS) generation (41). Thus, UQCR10 could mediate the effects of m^7^G-modified, tRNA-derived tRF-Lys-CTT on adipose thermogenesis. Moreover, reduced Uqcr10 expression decreases mitochondrial mass and lowers mitochondrial membrane potential in brown adipocytes. Together, these findings indicate that tRF-Lys-CTT may modulate thermogenic activation in adipocytes by regulating Uqcr10 expression.

In summary, this study broadens the functional understanding of tRNA epitranscriptomic modifications and noncoding RNA fragments in metabolic regulation and identifies post-transcriptional RNA silencing as a central mechanism controlling adipose thermogenesis. Moreover, it highlights novel molecular targets and supplies a theoretical foundation for manipulating adipose thermogenic capacity and for intervening in obesity and related metabolic syndrom.

## Experimental procedures

### Ethics approval and consent to participate

All experimental animals were ethically approved by the Animal Protection and Ethics Committee of Sichuan Agricultural University (Chengdu, China, Approval No. 20250402), and all procedures adhered to the welfare and ethical standards set by the university's Animal Management Committee.

### Animal experiments

Ten male and 30 female C57BL/6 mice (7–8 weeks old) were housed under identical conditions with ad libitum access to food and water. There were no significant differences in adipose tissue between the male and 30 female mice. The mice were randomly assigned to two groups: a room-temperature control group (n = 15) and a cold-exposure treatment group (n = 15), with 2 to 3 animals per cage. The control group was maintained at 22 to 26 °C. The cold-exposure group underwent a progressive short-term cold protocol at 4 °C: an initial 3 h exposure followed by 12 h at room temperature, a second 6 h exposure followed by another room-temperature recovery, and a final acute 48 h cold exposure. Aside from ambient temperature, all husbandry conditions were identical between groups. During model establishment, body weight and food intake were recorded daily, and bedding was changed daily. At the end of the protocol, mice were euthanized by cervical dislocation, and inguinal white adipose tissue (iWAT), epididymal white adipose tissue (eWAT), and interscapular brown adipose tissue (BAT) were collected; half of each tissue was stored at −80 °C and the other half was fixed in 4% paraformaldehyde and in electron microscopy fixative, respectively.

To generate a METTL1 knockdown mouse model, the CRISPR/Cas9 genome-editing system was used to specifically target the METTL1 gene. Among the five known transcripts at the METTL1 locus, the region encompassing exon 3 through exon 6 of the METTL1-201 transcript (ENSMUST00000006915.14) was chosen for deletion. This targeted segment comprises 551 base pairs of coding sequence, and its removal is expected to impair protein function. The CRISPR/Cas9 components were introduced into fertilized zygotes derived from C57BL/6J mice *via* microinjection, and the resulting embryos were then transferred into pseudopregnant foster females. Founder (F0) animals were genotyped using PCR and sequencing to verify the intended editing events. Subsequently, confirmed F0 mice were mated with wild-type C57BL/6J mice to generate a stable F1 generation carrying the desired METTL1 modification.

### Cell culture, treatment, and transfection

Mouse embryonic fibroblasts (C3H10T1/2) were obtained from the National Infrastructure of Cell Line Resource (NICR) and maintained in DMEM (12491015, Gibco) supplemented with 10% (v/v) fetal bovine serum and 1% (v/v) penicillin/streptomycin at 37 °C in a humidified incubator with 5% CO2. According to the experimental protocol, C3H10T1/2 cells were seeded into 12-well plates and cultured until 100% confluence, followed by an additional 48 h. Upon completion of contact inhibition, the proliferation medium was replaced with an induction differentiation medium containing insulin (20 μM), 3-isobutyl-1-methylxanthine (0.5 mM), dexamethasone (1 μM), 3,3′,5-triiodo-L-thyronine (Triiodothyronine, T3, 3 nM), indomethacin (125 μM), and rosiglitazone (5 μM). After 2 days of induction, the medium was switched to a maintenance medium containing only insulin (20 μM), 3,3′,5-triiodo-L-thyronine (3 nM), and rosiglitazone (5 μM). The maintenance medium was changed every 2 days, and mature C3H10T1/2 brown adipocytes were typically obtained within 8 to 10 days after induction.

To establish cellular models, cells were transfected with tRF mimics, a mimic negative control (Mimic-NC), siRNAs, and an siRNA negative control (si-NC) synthesized and designed by GenePharma. Transfections were carried out with Hieff Trans Universal Transfection Reagent (Yeasen Bio) following the manufacturer's instructions. All sequences are provided in Table S1 (Supplementary).

Forskolin activates the cAMP signaling pathway in treated cells, thereby modeling cellular thermogenic activation. A 10 mM forskolin stock solution is prepared; after cells have completed induced differentiation, replace the medium with maintenance medium containing forskolin (10 μM). Cells can be harvested 6 to 12 h after treatment.

### tsRNA & tRF sequencing

Adipose tissues were collected from mice and total RNA was extracted. Modified groups were removed using an RNA pretreatment kit, and libraries were prepared with the Small RNA Library Prep Kit (Illumina). After library quality control, sequencing was carried out on an Illumina NovaSeq 6000 platform. Differentially expressed tsRNAs/tRFs were identified using thresholds of log2 fold change > 1 and adjusted *p* < 0.05.

### Untargeted metabolomics sequencing

Brown adipose tissue (BAT) was collected from mice subjected to both cold exposure and control treatments. Residual blood was removed using pre-cooled deionized water. Following collection, adipose tissue samples were rapidly quick-frozen in liquid nitrogen and subsequently stored at −80 °C until metabolite extraction. An appropriate amount of the frozen and ground tissue sample was weighed, and pre-cooled extraction solvent was added. The mixture was vortexed thoroughly and subjected to low-temperature ultrasonic extraction. After extraction, the samples were centrifuged at 4 °C for 15 min at 12,000 rpm, and the supernatant was collected for further analysis. Sample extraction and detection were performed by Novogene Co., Ltd. Chromatographic separation was achieved using the Waters ACQUITY UPLC I-Class system. Mobile phases A and B were prepared according to the ionization mode: in positive ion mode, 0.1% formic acid aqueous solution (A) and methanol (B) were utilized; in negative ion mode, a 5 mM ammonium acetate aqueous solution (A) and methanol (B) were employed. The following gradient elution program was implemented for separation: 0 to 1.5 min, 98% A and 2% B; 1.5 to 3 min, 15% A and 85% B; 3 to 10 min, 100% B; and 10 to 12 min, 98% A and 2% B.

The Q Exactive HF-X mass spectrometry platform, known for its ultra-high resolution and sensitivity, was utilized in this study. Equipped with an electrospray ionization (ESI) source, the mass spectrometer operated at a spray voltage of 3.5 kV. The sheath gas flow rate was maintained at 35 psi, while the auxiliary gas flow rate and heater temperature were set at 10 L/min and 350 °C, respectively. The ion transfer tube temperature (Capillary Temp) was held constant at 320 °C, and the S-lens RF level was adjusted to 60. Both positive and negative ion modes (Polarity: positive, negative) were employed to ensure comprehensive analysis of metabolites with varying polarities. For MS/MS secondary scanning, the scan range spanned from m/z 100 to 1500, utilizing a data-dependent scan mode to improve sensitivity and selectivity in the analysis.

Raw data were preprocessed and converted using the metaX metabolomics software, and the resulting dataset was analyzed by principal component analysis (PCA) and partial least squares discriminant analysis (PLS-DA) to generate VIP scores for each metabolite. Between-group differences were evaluated with Student’s *t* test (reported as *p* values), and fold change (FC) values were calculated. Differential metabolites were selected according to VIP, *p* value, and FC thresholds. Finally, functional annotation and pathway enrichment of the differential metabolites were carried out using the KEGG database.

### Dual luciferase reporter assay

The 3′UTR of the target gene or its mutant variant was cloned into a luciferase reporter vector and transfected into HEK293T cells. After 24 h, cells were lysed and assayed with a dual-luciferase assay kit (Promega) according to the manufacturer’s instructions. Firefly and Renilla activities were measured independently, and the Firefly/Renilla (Fluc/Rluc) ratio was calculated for normalization.

### Quantitative reverse transcription PCR (qRT-PCR)

Total RNA was extracted with TRIzol reagent. Small RNAs were reverse-transcribed using stem-loop RT primers according to the Mir-X miRNA First-Strand Synthesis Kit protocol (Takara Bio). mRNAs were reverse transcribed with the PrimeScript FAST RT reagent Kit including gDNA Eraser (Takara Bio) following the manufacturer’s instructions. qPCR was performed with SYBR Green Master Mix (Takara Bio) on a real-time instrument. U6, GAPDH, or β-actin served as reference genes, and relative expression was calculated by the 2^-ΔΔCt^ method. Primer sequences are listed in Table S2 (Supplementary Information).

### Western blot analysis

Cells were lysed and total protein was extracted in SDS lysis buffer (P0013G, Beyotime Bio) supplemented with 1% PMSF (ST2573, Beyotime Bio). Lysates were clarified by centrifugation at 12,000×*g* for 10 min, and the supernatants were mixed with 5× SDS loading buffer and denatured at 95 °C for 10 min. Proteins were resolved on 4%-12% SDS-PAGE gels and transferred to PVDF membranes (Millipore). Membranes were blocked with blocking buffer (PD206, Oriscience) for 10 to 20 min, incubated with primary antibodies overnight at 4 °C, and then incubated with species-appropriate secondary antibodies for 1 h. Bands were detected with ECL (Share bio, SB-WB004) and quantified using ImageJ. Detailed antibody information is provided in Table S3 (Supplementary).

### Dot blot

Cells were collected and washed once with chilled PBS, after which total RNA was extracted using the Trizol method. RNA quality and concentration were assessed, and concentrations were normalized across samples to an appropriate level. Aliquots of 500 ng-1 μg RNA were denatured in a 65 °C metal bath for 5 min and immediately placed on ice for 2 min. RNA samples were then uniformly spotted onto the front side of the membrane. After air-drying, the membrane was UV-crosslinked at 120 mJ/cm^2^ for 5 min to covalently immobilize the RNA. The membrane was blocked for 1 h in blocking buffer (TBST or PBST containing 5% nonfat milk) and incubated overnight at 4 °C with the primary antibody. The membrane was then incubated with the appropriate species-matched secondary antibody for 1 h. Signal was developed by ECL detection. After development, the nylon membrane was washed twice, stained at room temperature with methylene blue for 5 min, and scanned for image acquisition. Densitometric analysis was performed using ImageJ software. Detailed antibody information is listed in Table S2 (Supplementary Information).

### Mitochondrial membrane potential JC-1 staining and Mito-Tracker staining

Cells for assay were collected, washed once with PBS, and incubated with JC-1 working solution prepared according to the manufacturer's instructions (C2006, Beyotime Bio). After incubation at 37 °C for 30 min, cells were washed twice with staining buffer, resuspended in an appropriate volume of culture medium, and imaged by fluorescence microscopy (Olympus). Mito-Tracker staining was performed in the same manner using the Mito-Tracker Red CMXRos detection kit (C1035, Beyotime Bio).

### RNA immunoprecipitation (RIP)

Cells were lysed in NP-40 buffer supplemented with RNase inhibitor (100 U RNase Inhibitor, 2313B, Takara) and protease inhibitors (1× cOmplete Protease Inhibitor Cocktail, Roche). Lysates were clarified, and protein concentrations were measured with the BCA assay kit (P0010S, Beyotime). Supernatants were incubated with 3 μg of primary antibody or control IgG at 4 °C with rotation overnight. The antibody-lysate complexes were then captured with Dynabeads Protein G (Thermo Scientific) for 1 to 2 h. Finally, RNA co-purified with the protein complexes was extracted, purified, and analyzed by qRT-PCR.

### Immunofluorescence (IF)

Harvest cells after induced differentiation and after transfection or drug treatment. Wash the cells with 1× PBS, then fix them in 4% paraformaldehyde at room temperature for 10 min. Permeabilize the fixed cells with 0.1%-0.3% Triton X-100. Block in PBS containing 10% bovine serum albumin (BSA) or goat serum for 1 h, then incubate overnight at 4 °C with the prepared primary antibody working solution. Incubate the cells with the fluorescent secondary antibody at room temperature for 1 h. Counterstain nuclei with DAPI. Observe and capture images using a fluorescence microscope.

### Ribo-seq and RNA-seq

Fresh adipose tissue was collected from mice, snap-frozen in liquid nitrogen, and stored at −80 °C. Samples designated for analysis were transported under ultra-low-temperature conditions to Guangzhou Epigenetics Biotech Co., Ltd for Ribo-seq and RNA-seq. A portion of each tissue sample was processed with TRIzol reagent (Takara) to extract total RNA for RNA-seq library preparation and sequencing. The remaining tissue was homogenized and lysed to purify ribosome-protected fragments, which were then used for library construction and sequencing.

### Bodipy staining

Seed the appropriate number of cells into 12-well culture plates, then perform transfection or apply drug treatments according to the experimental design. After treatment, discard the culture medium and wash the cells twice with 1× PBS. Remove the PBS and add an appropriate volume of 4% paraformaldehyde; fix the cells at room temperature for 20 min. After fixation, remove the fixative and wash the cells twice with 1× PBS. Remove the PBS and add a suitable volume of Staining Solution (prepared according to the kit instructions), then incubate in the dark at room temperature for 20 min. After staining, wash three times with 1× PBS and examine and photograph the cells using a fluorescence microscope.

### Oil Red O staining

After transfection or drug treatment, wash cells twice with 1× PBS. Remove the PBS and add 500 μl of 4% paraformaldehyde to fix the cells for 20 min. Prepare the Oil Red O working solution by mixing the stock and diluent at a 3:2 (v/v) ratio. After fixation, remove the fixative and wash the cells twice with PBS. Add an appropriate volume of the Oil Red O working solution and incubate at room temperature for 10 to 20 min. After staining, remove the working solution and add an appropriate volume of the kit-provided staining wash solution; let stand for 30 s. Carefully aspirate the wash solution with a pipette and rinse once with 1× PBS. Observe the cells and capture images under a microscope. After image acquisition, discard the PBS and add an equal volume of isopropanol to each well to extract intracellular Oil Red O dye with gentle agitation at room temperature. Transfer the extract to a microplate and measure absorbance at 510 nm using a multimode plate reader.

### Statistical analysis

All data are presented as mean ± standard deviation (mean ± SD). Statistical analyses were performed using GraphPad Prism 9.0 (GraphPad Software). Group comparisons used unpaired Student’s t tests with Bonferroni correction for multiple comparisons. The threshold for significance was *p* < 0.05. Notation: ns, not significant; ⁎, *p* < 0.05; ⁎⁎, *p* < 0.01; ⁎⁎⁎, *p* < 0.001; ⁎⁎⁎⁎, *p* < 0.0001.

## Consent for publication

All authors have thoroughly reviewed this manuscript and kindly request its exclusive consideration for publication in Journal of Biological Chemistry.

## Data availability

The data that support the findings of this study are obtained in this article. The RNA sequencing raw data can be obtained in the NCBI database under the accession numbers: PRJNA1501680.

## Supporting information

This article contains supporting information.

## Conflict of interest

The authors declare that they have no conflicts of interest with the contents of this article.
