# Supplementary material for: An m7G tRNA-derived fragment mediated thermogenic activation of brown adipose tissue through post-transcriptional repression of Uqcr10
Source: J Biol Chem. 2026 Oct 14;302(12):113640. doi: 10.1016/j.jbc.2026.113640 (PMC13769676; doi:10.1016/j.jbc.2026.113640)

Raw image files of full gels or western blots

Figure 1

Figure 1B

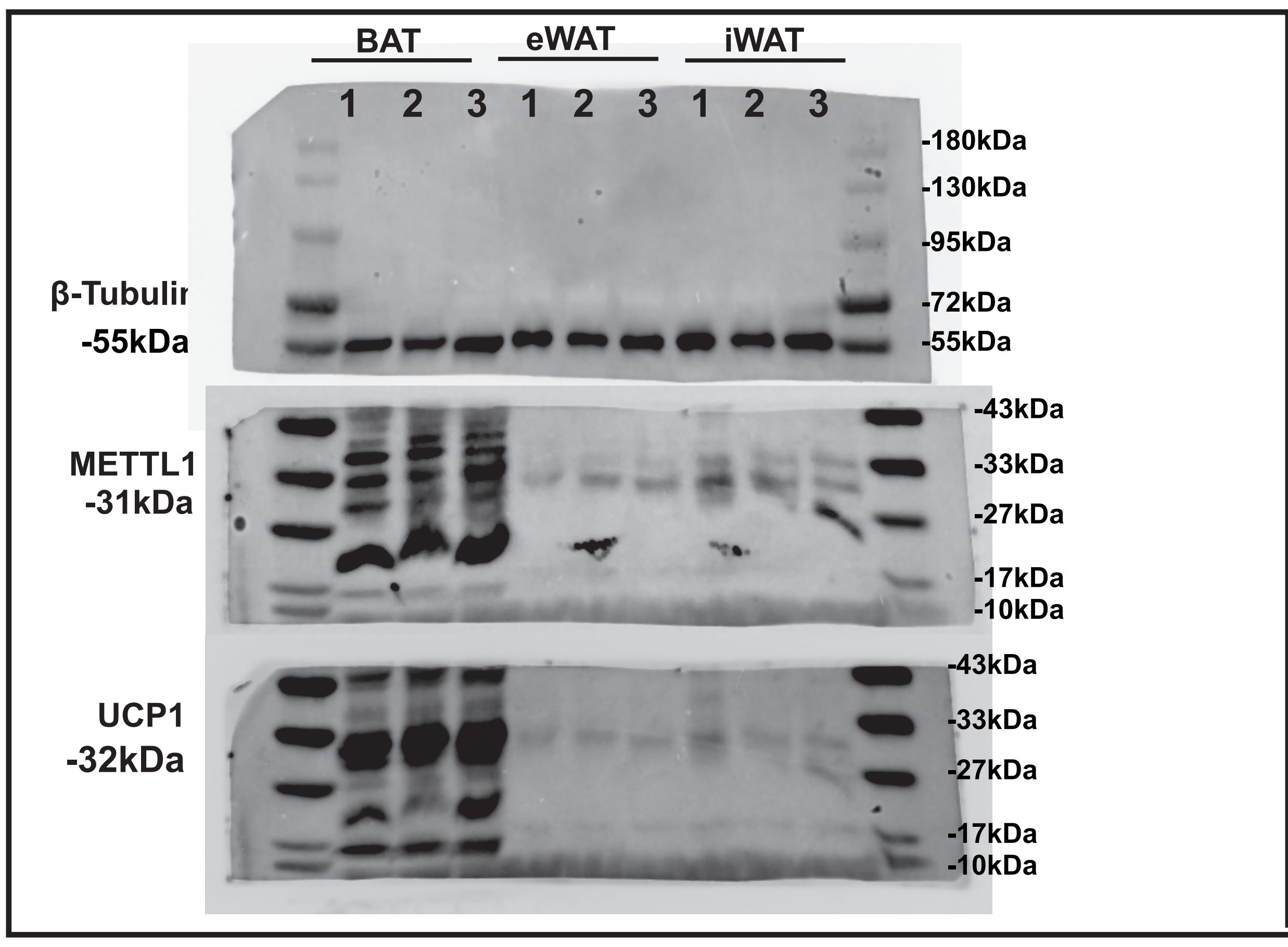

Figure 1C

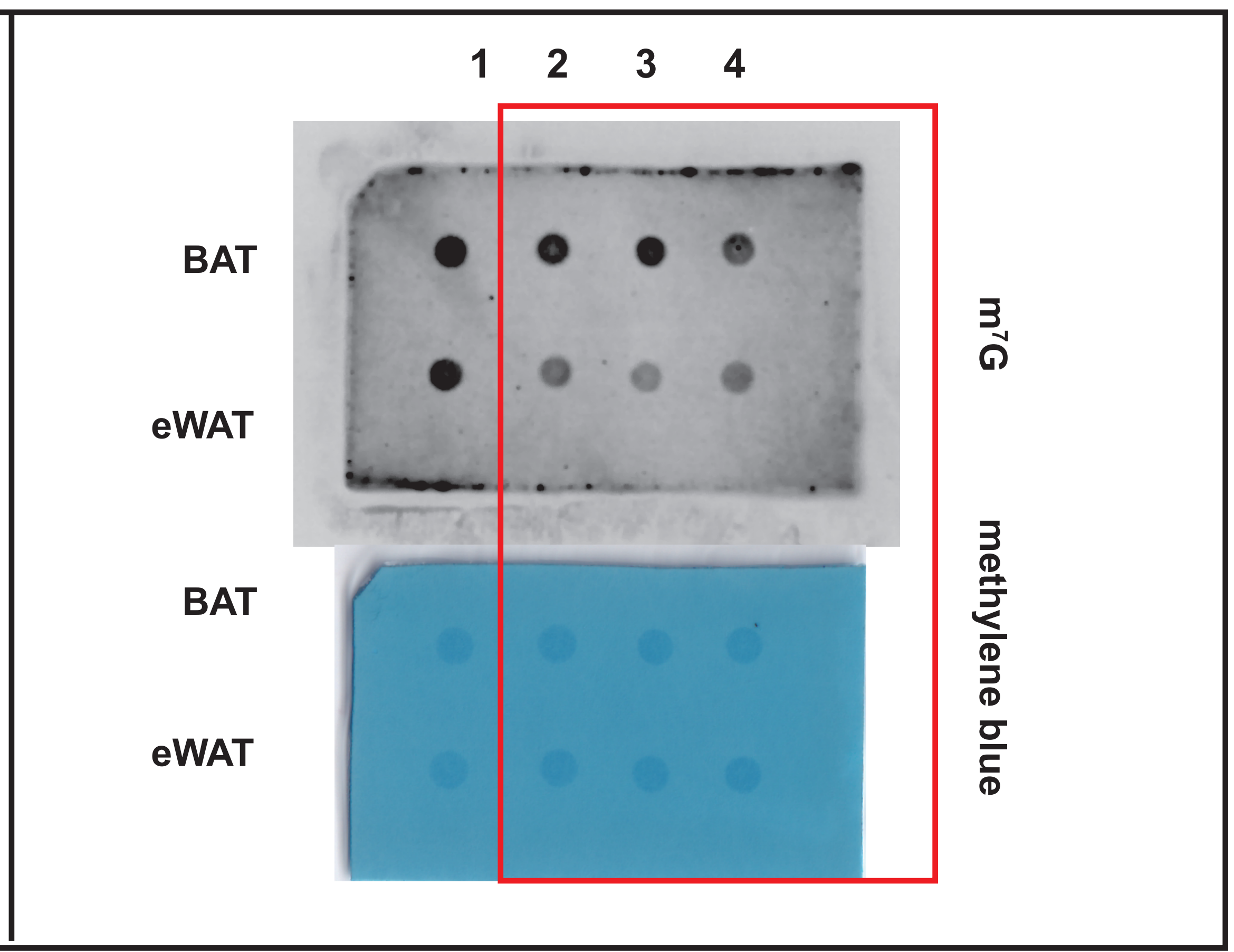

Figure 1O

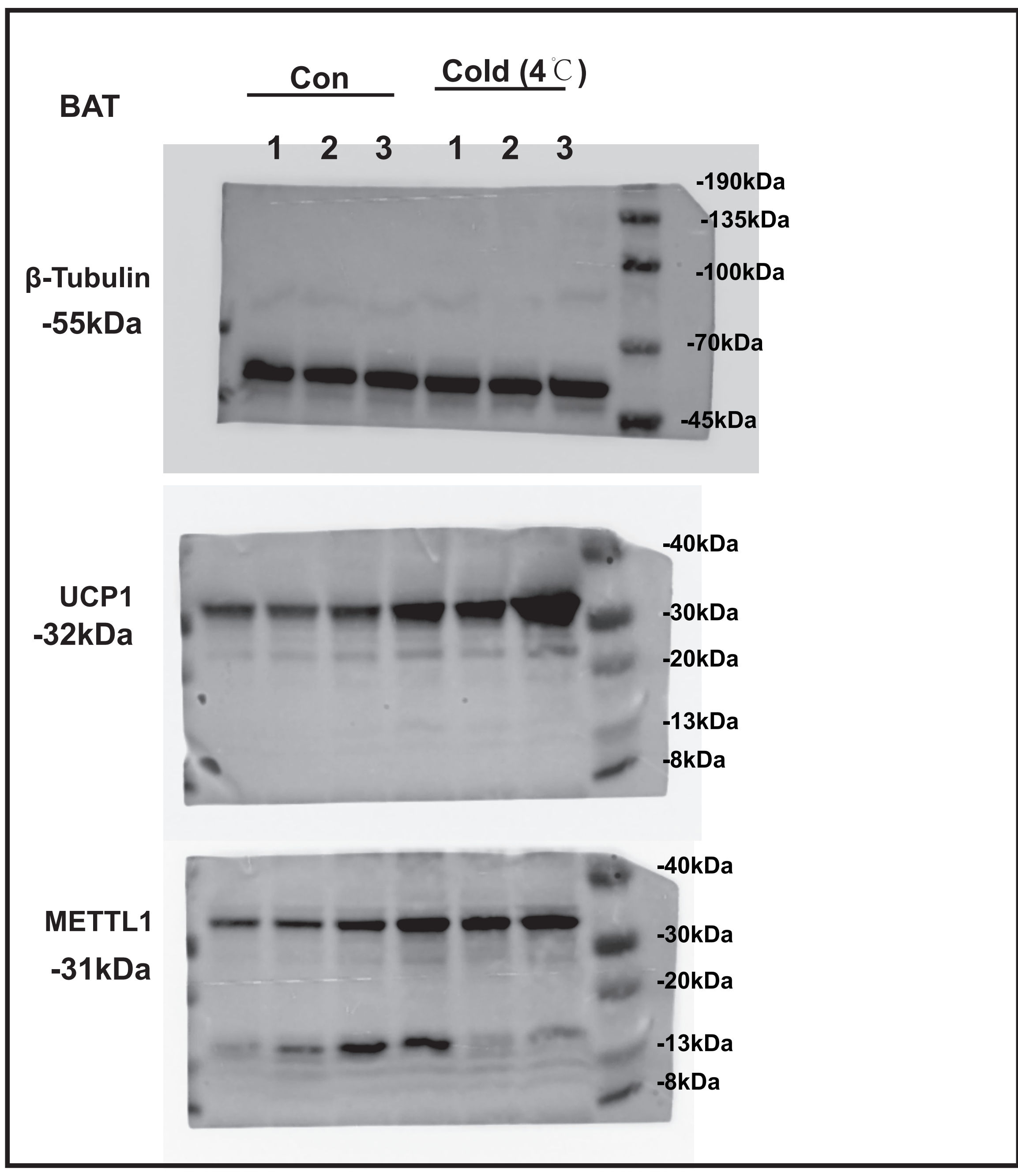

Figure 1P

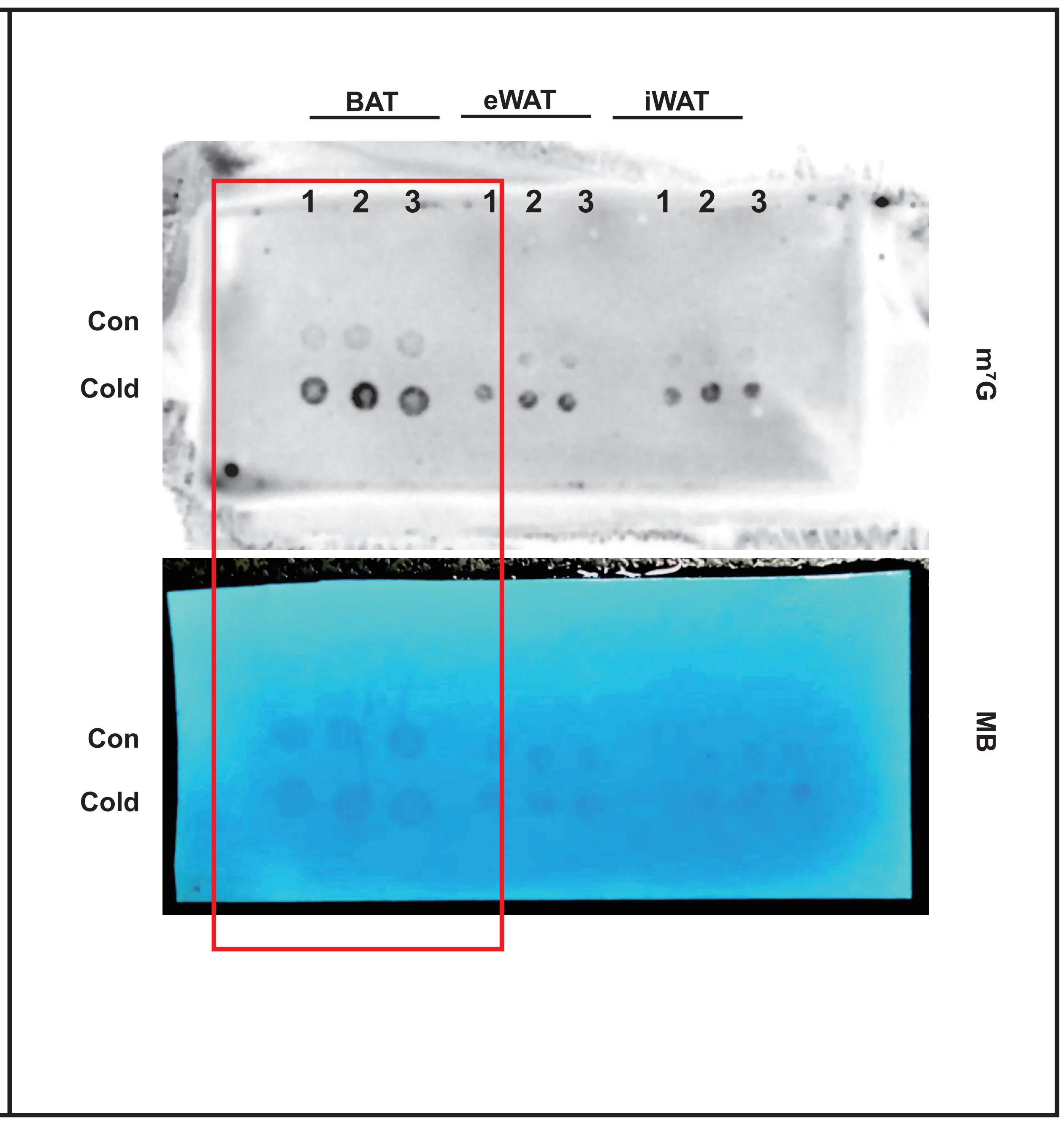

Figure 3

Figure 3B

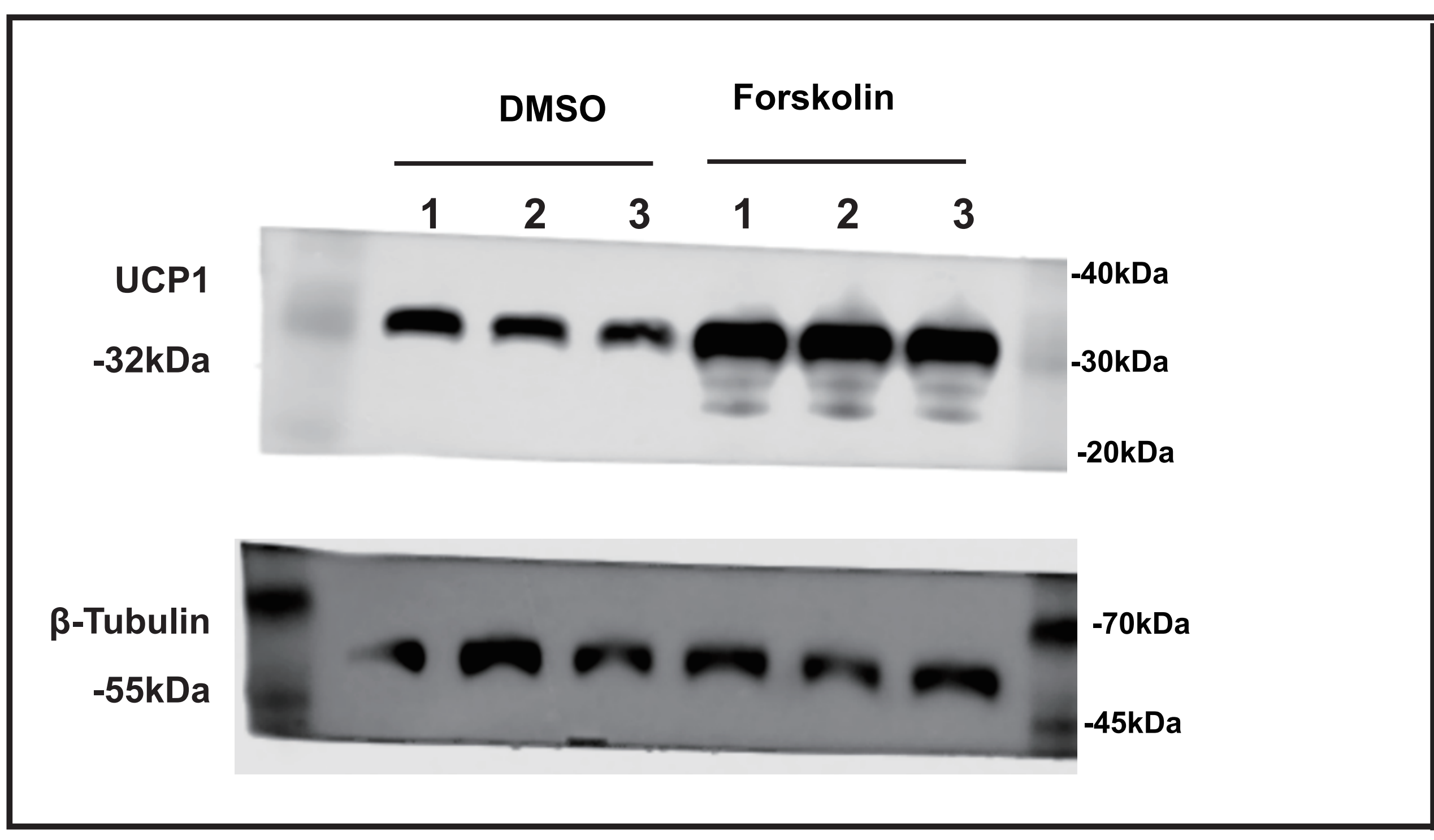

Figure 3J

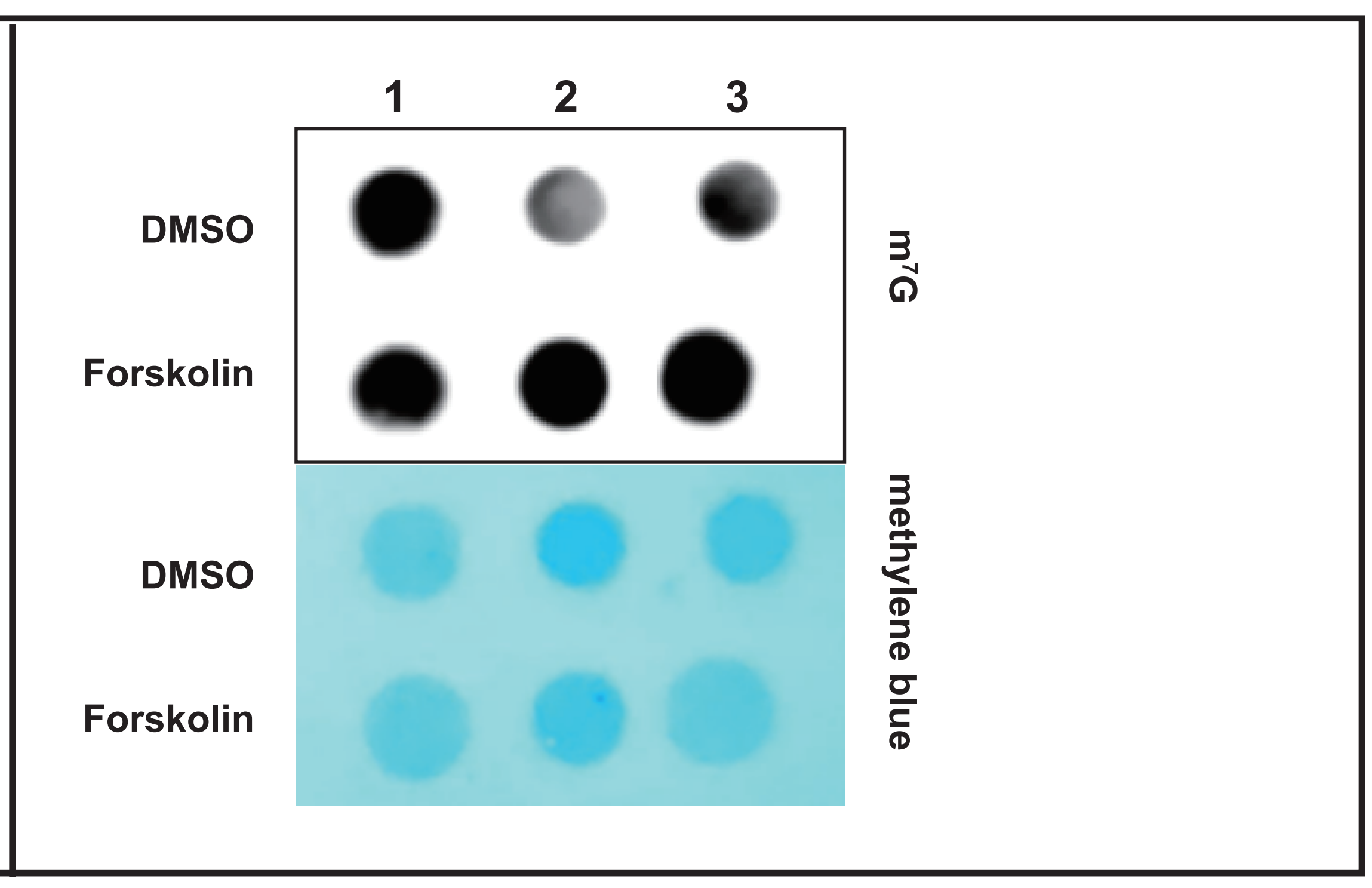

Figure 3J

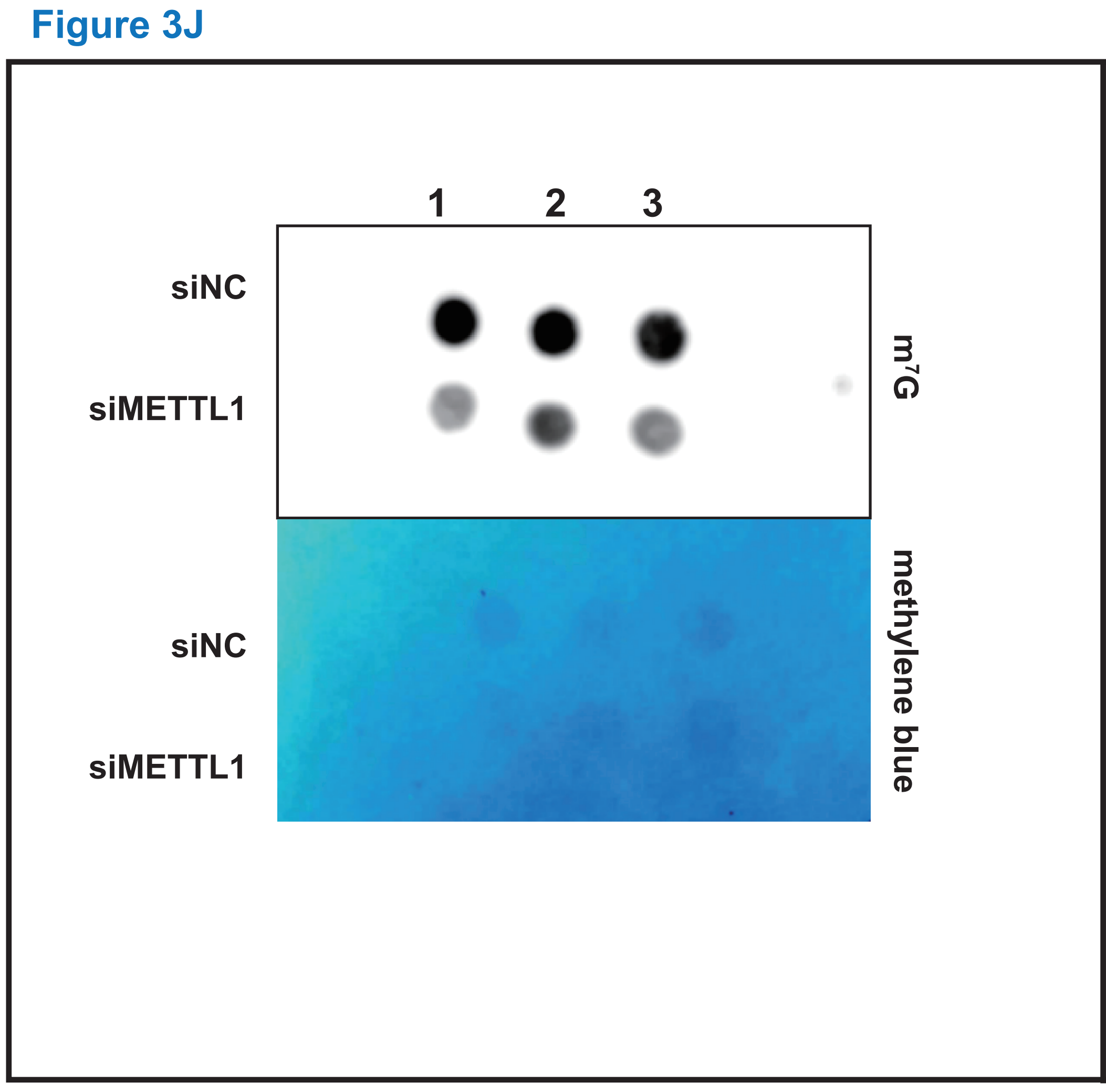

Figure 3N

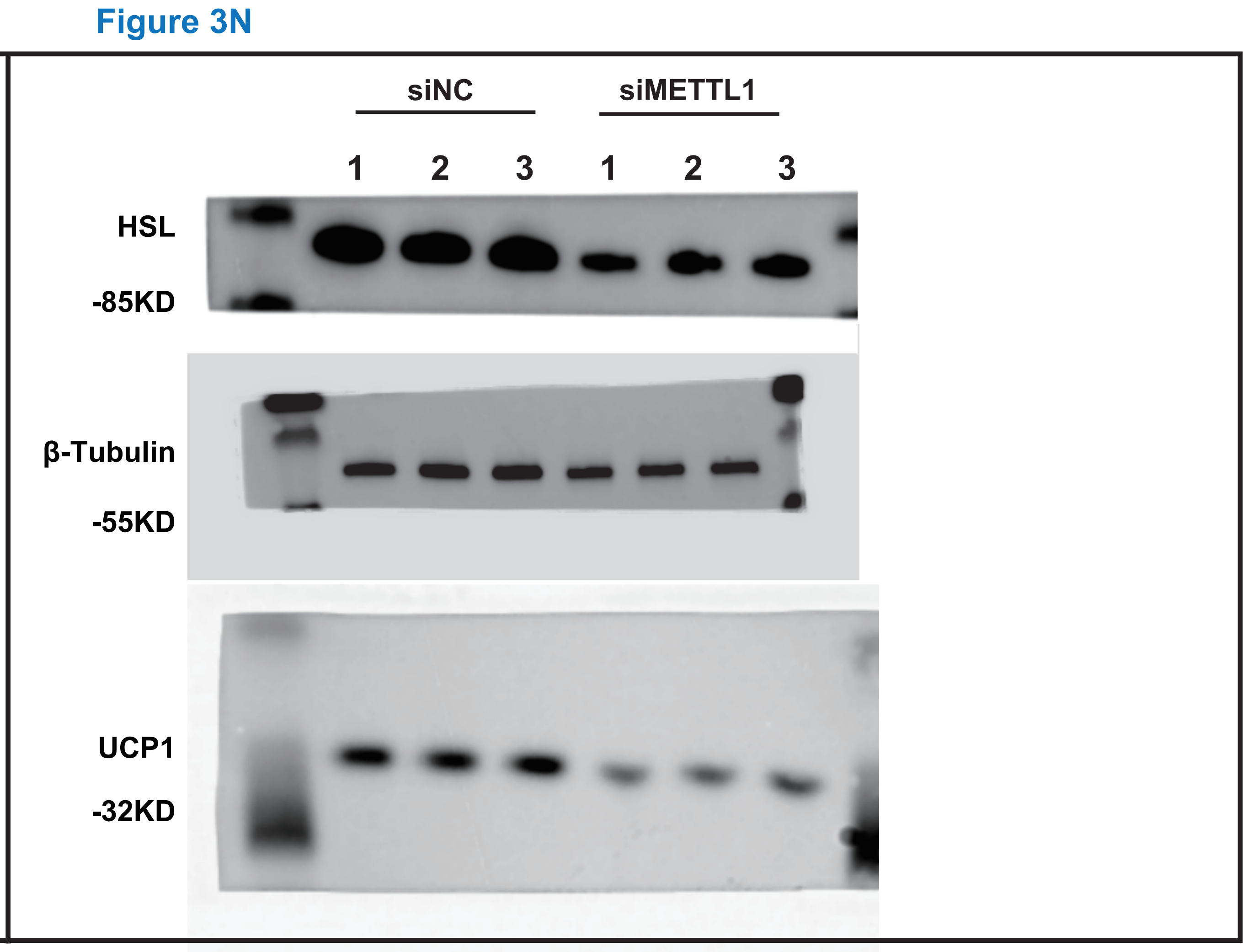

Figure 3

Figure 3R

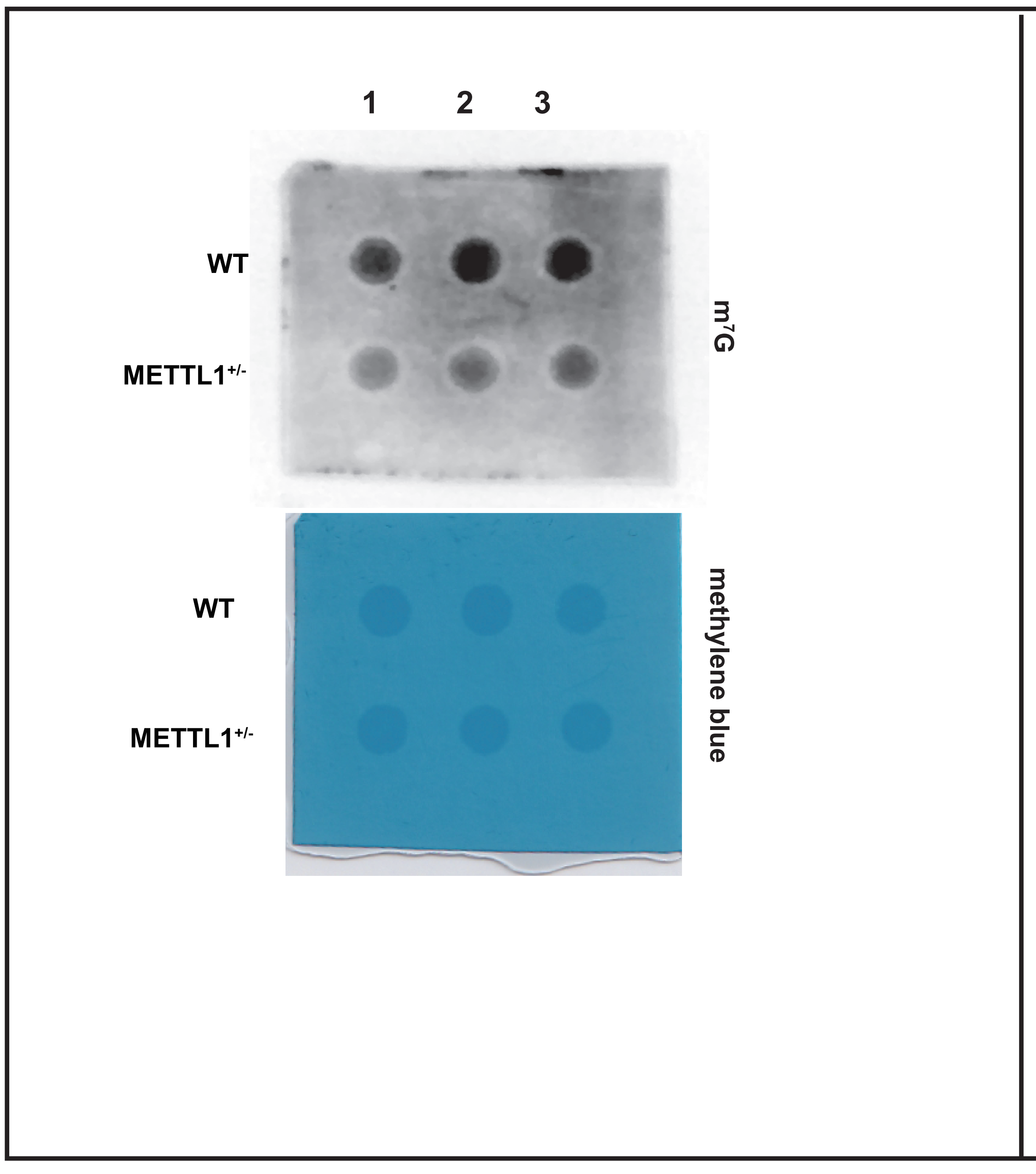

Figure 5

Figure 5E

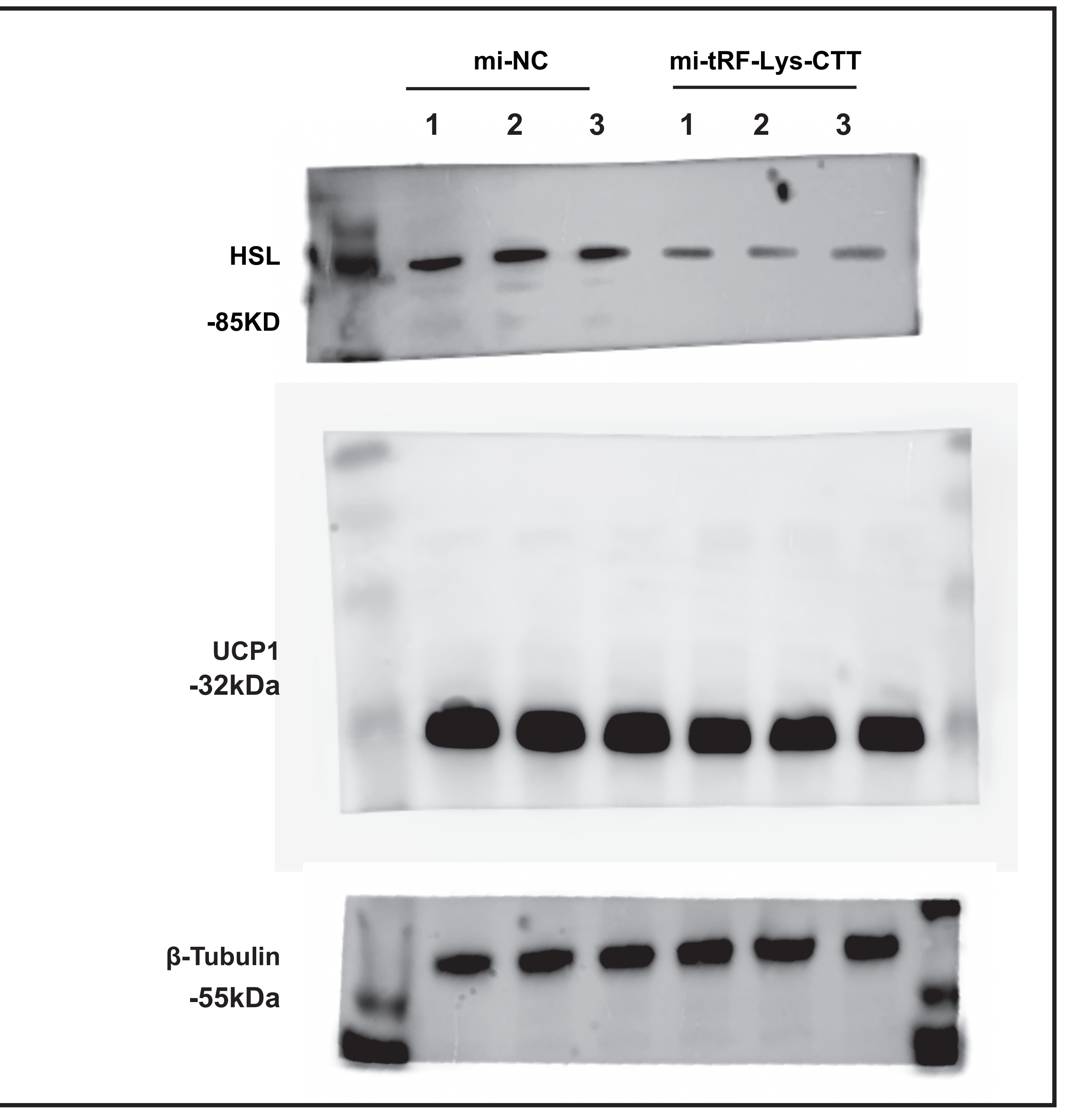

Figure 7

Figure 7A (left)

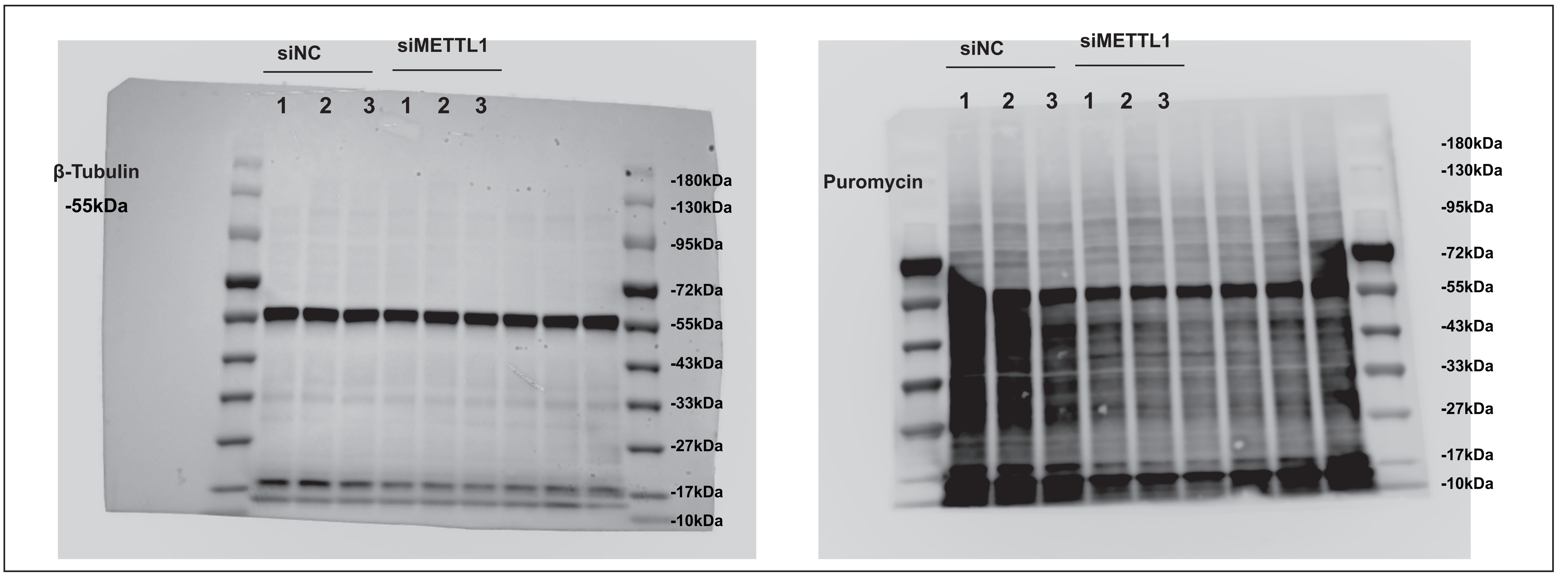

Figure 7A (right)

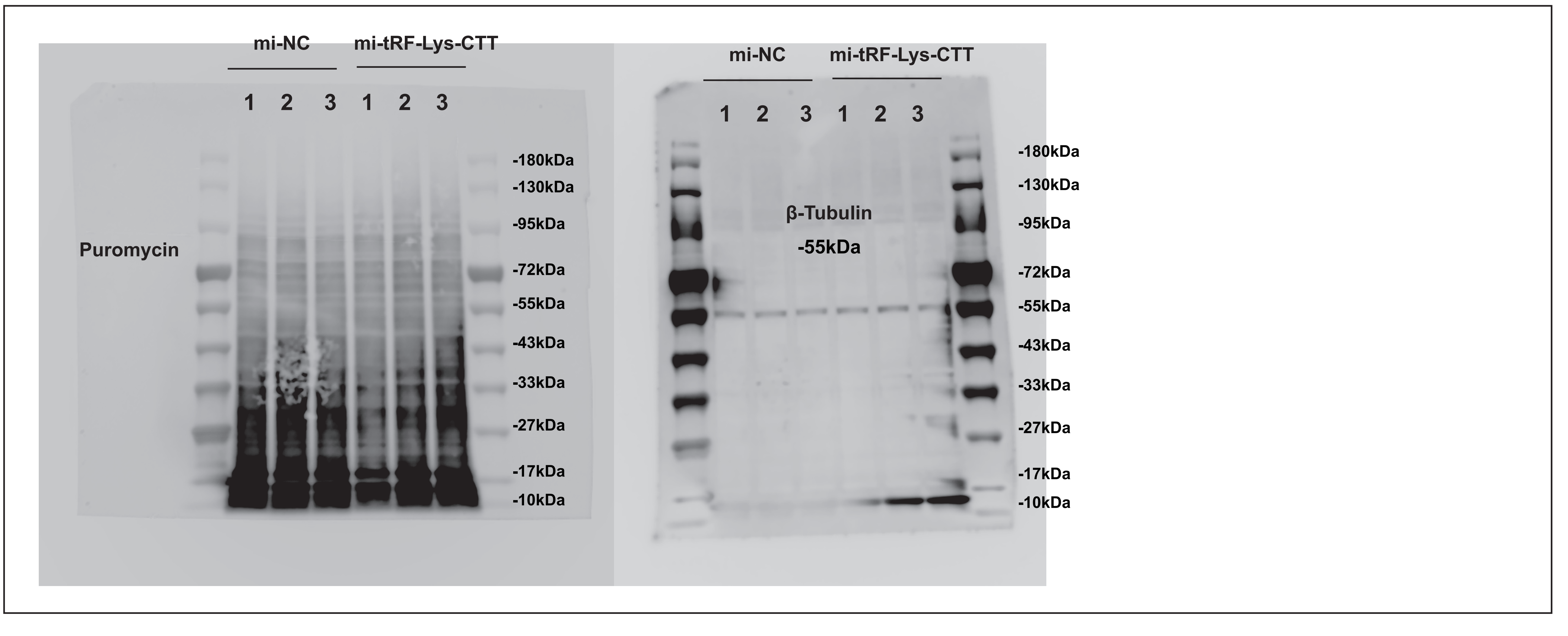

Figure 7F

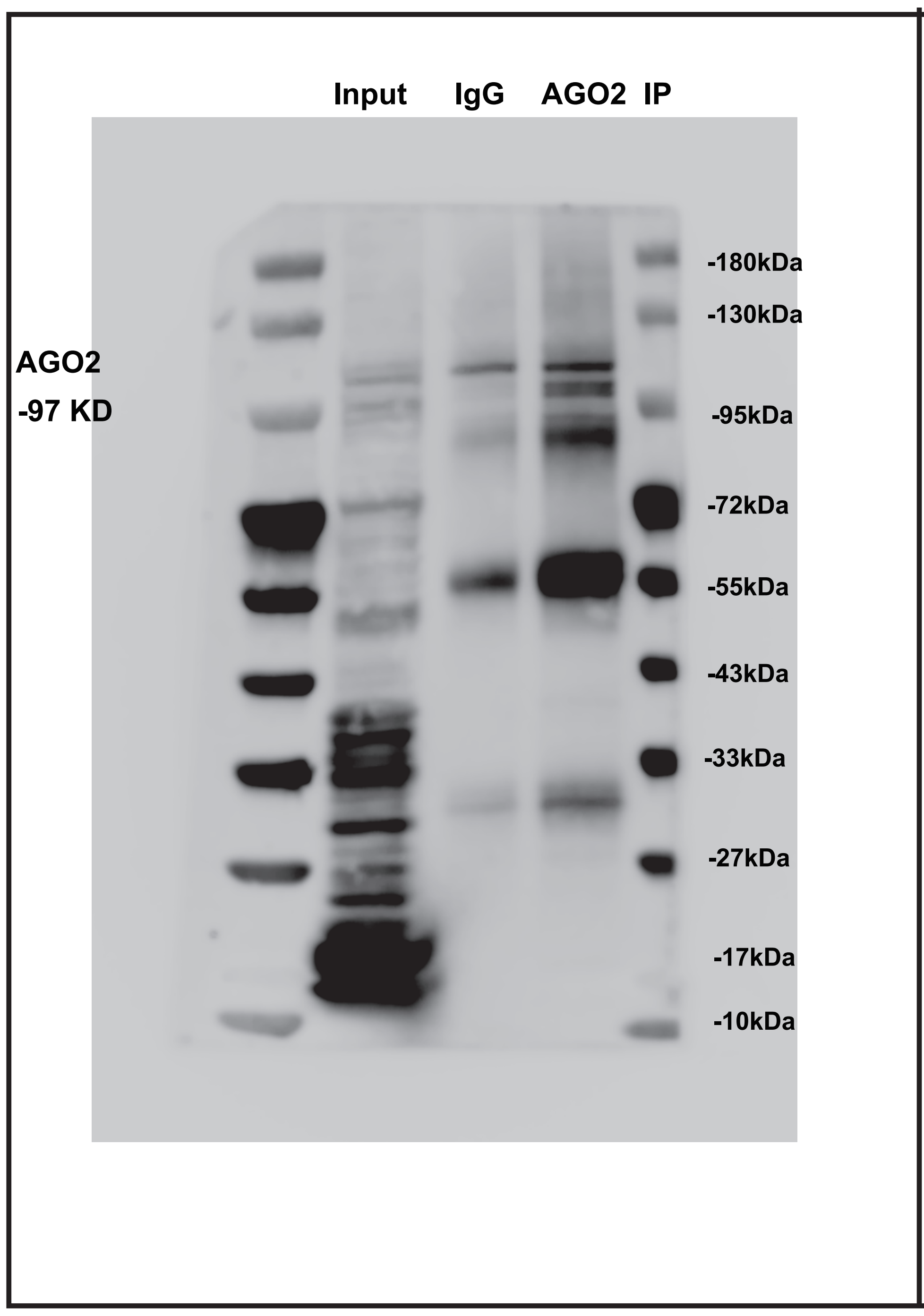

Figure 7G

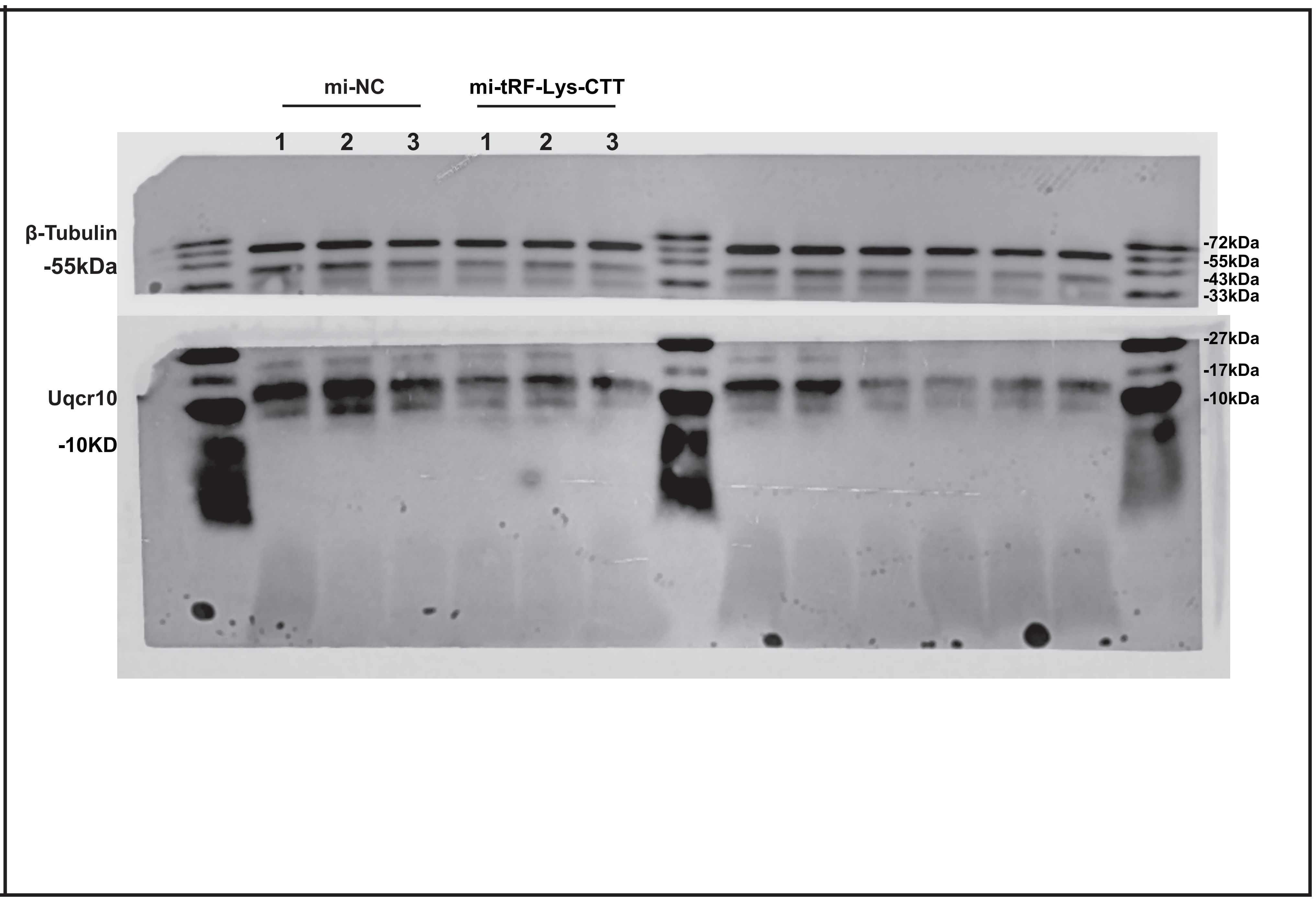

Figure 7I

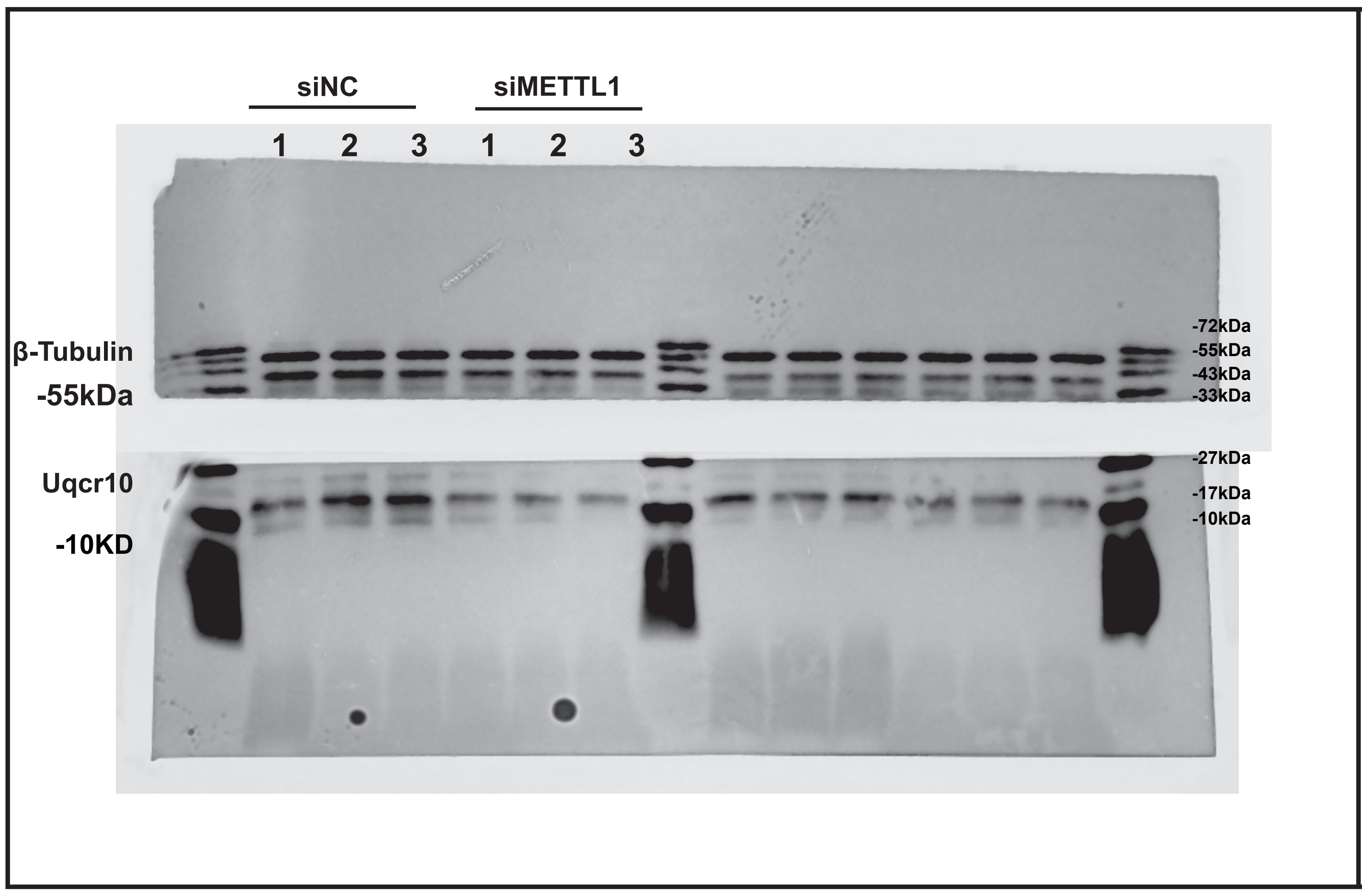

Figure 7K

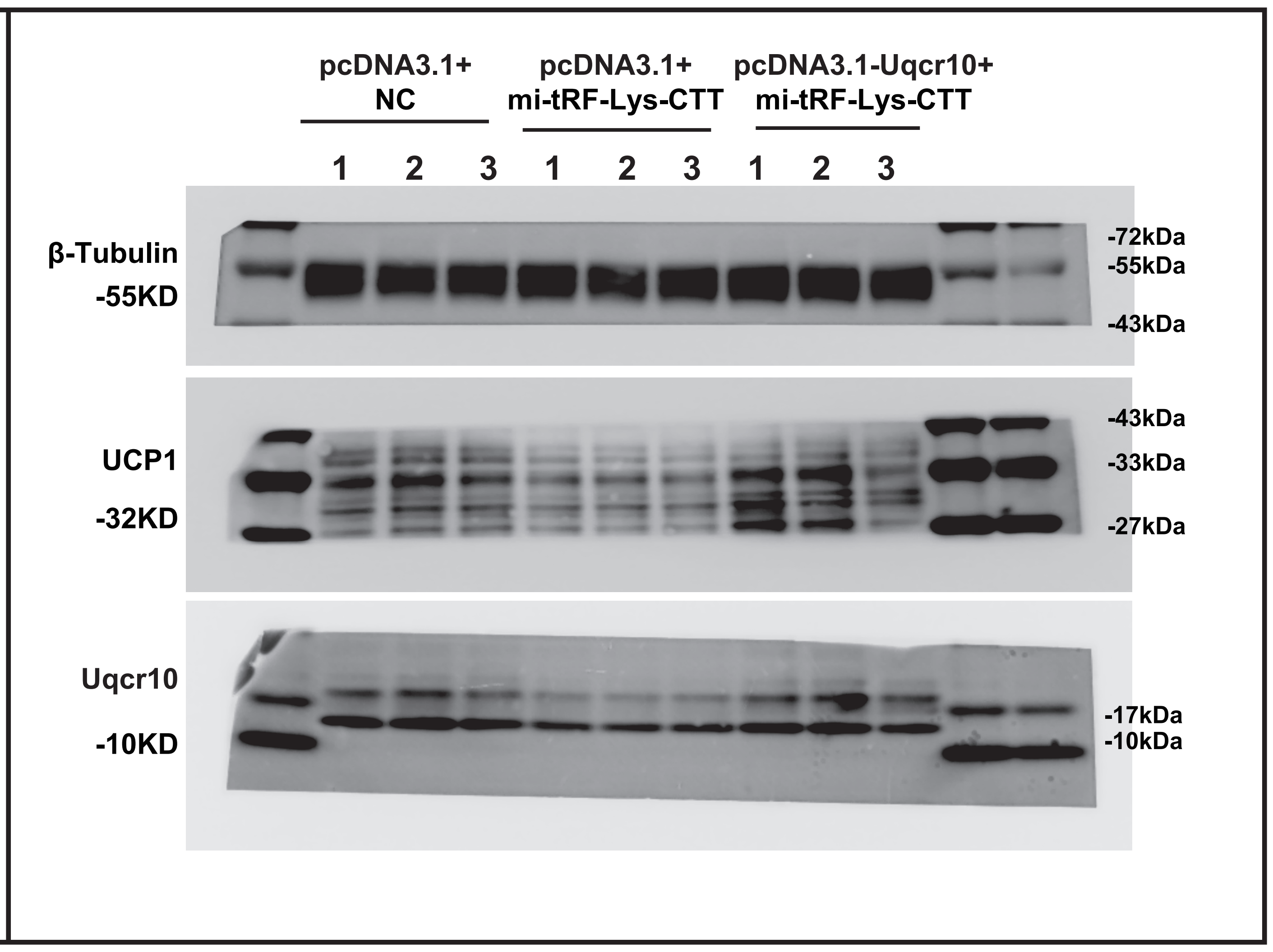

Figure 7L

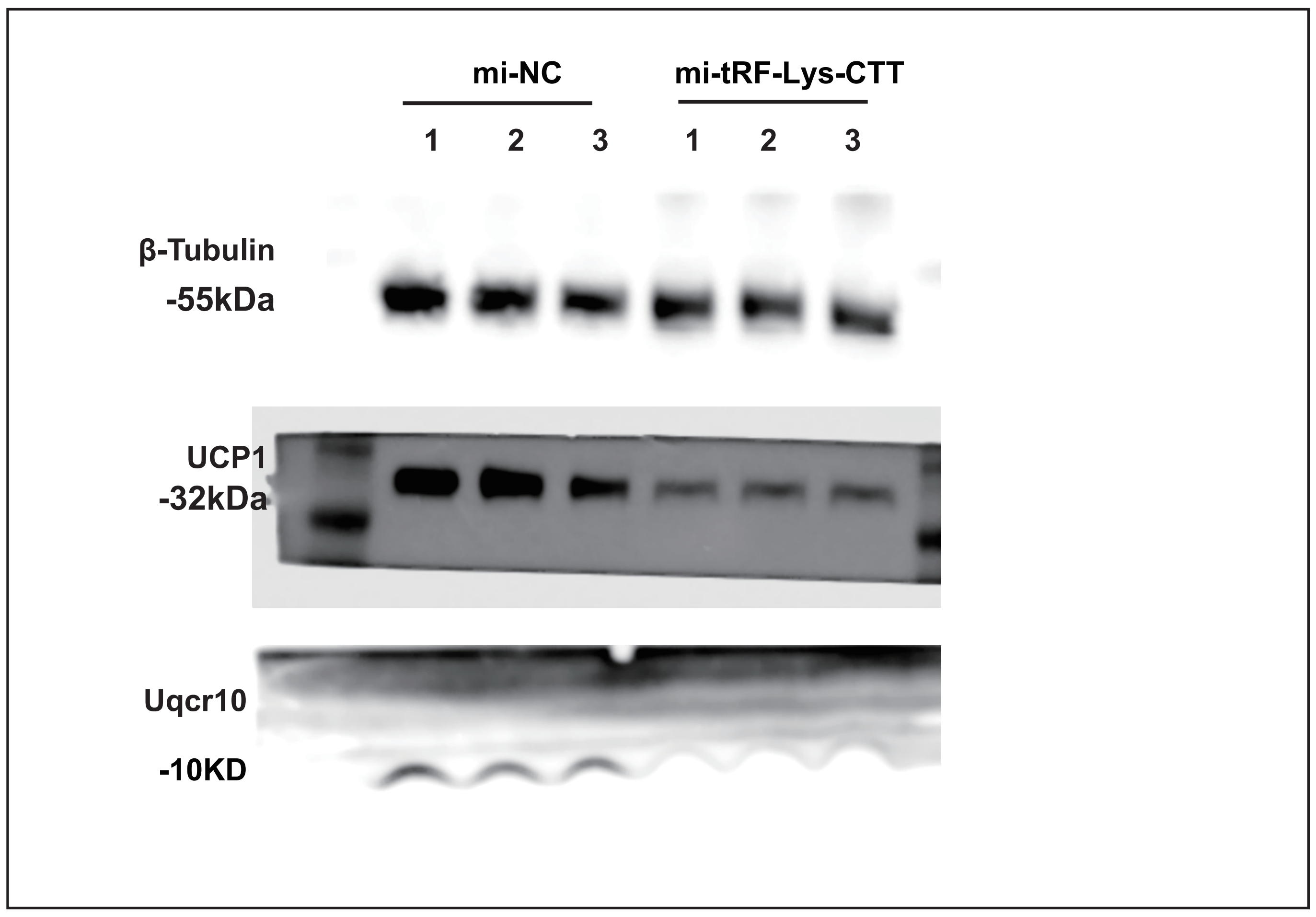

Supplement: Supplementary file 1 [file mmc1.pdf]
