# Supplementary material for: An m7G tRNA-derived fragment mediated thermogenic activation of brown adipose tissue through post-transcriptional repression of Uqcr10
Source: J Biol Chem. 2026 Oct 14;302(12):113640. doi: 10.1016/j.jbc.2026.113640 (PMC13769676; doi:10.1016/j.jbc.2026.113640)
Supplement: Supplementary file 2 [file mmc2.docx]

**Supporting Information**

**An m^7^G tRNA-derived fragment mediated thermogenic activation of brown adipose tissue through post-transcriptional repression of Uqcr10**

Xue Zhao *et al.^*^*

*Corresponding author: Li Zhu, zhuli@sicau.edu.cn; Linyuan Shen, shenlinyuan@sicau.edu.cn

**This PDF file includes:**

Supplementary Text

Figures S1 to S4

Tables S1 to S3

**Fig. S1 Metabolic differences during brown adipose tissue thermogenic activation induced by cold exposure.** (A-D) Partial least squares discriminant analysis (PLS-DA) score plots in positive ion (A) and negative ion (C) modes, and PLS-DA permutation tests in positive ion (B) and negative ion (D) modes. (E-F) GSEA enrichment analysis of pathways enriched by differential metabolites in positive (E) and negative (F) ion modes.

**Fig. S2 METTL1 deficiency impairs Forskolin-induced thermogenic activation.** (A) immunofluorescence images showing UCP1 expression (red) in brown adipocytes treated with Dmso, Forskolin, or Forskolin combined with si-METTL1. Nuclei were counterstained with DAPI (blue). (B) Quantification of the relative fluorescence intensity of UCP1 across the indicated experimental groups. (C) Mito-Tracker staining (red) showing mitochondrial abundance and morphology under the designated treatment conditions. Nuclei were counterstained with DAPI (blue). (D) Quantification of the relative Mito-Tracker fluorescence intensity. Data are presented as mean ± SD. Student’s t-test. * *p* < 0.05, *** *p* < 0.001.

**Fig**. **S3 RNA-seq analysis of genes during BAT thermogenic activation.** (A) Volcano plot displaying differentially expressed genes (DEGs) identified by RNA-seq (|log2FC| > 1, *P* < 0.05). (B-C) KEGG pathway enrichment analysis for downregulated (B) and upregulated (C) DEGs. (D-E) GO (Biological Process) enrichment analysis for downregulated (D) and upregulated (E) DEGs.

**Fig**. **S4** **tRF-Lys-CTT participates in the regulation of protein synthesis and modulates the expression of thermogenesis-related genes by targeting Uqcr10.** (A) A Venn diagram displays the intersection of four independent datasets used to identify downstream targets of the METTL1/tRF‑Lys‑CTT axis: bioinformatically predicted targets, differentially expressed proteins (DEPs) from proteomics, differentially expressed genes (DEGs) from transcriptomics, and genes with altered translational efficiency (TE). (B) RT‑qPCR quantification of Uqcr10, Slc25a20, and Acsl5 mRNA in BAT, eWAT, and iWAT. (C) qPCR measurement of Uqcr10, Slc25a20, and Acsl5 expression in forskolin‑activated C3H10T1/2 cells after si‑METTL1 transfection. (D) Predicted secondary structure of the tRF-Lys-CTT–Uqcr10 duplex generated with RNAhybrid. (E) Quantification Results for Uqcr10 and UCP1 Western Blot Bands after co-transfection with PcDNA3.1-Guf1 and tRF-Lys-CTT mimics. (F) Quantification Results for Uqcr10 and UCP1 following si-Uqcr10 transfection. n = 3. Student’s t‑test; data are mean ± SD. **p* < 0.05, ***p* < 0.01, ****p* < 0.001, *****p* < 0.0001; ns, not significant.

**Table S1.** Sequences of siRNA, mimic, inhibitor and probe used in this study.

| **Gene** | **Foward primer** | **Reverse primer** |
| --- | --- | --- |
| siMETTL1 | GGAACAUCUAGGCAGUUCATT | UGAACUGCCUAGAUGUUCCTT |
| siNC | UUCUCCGAACGUGUCACGUTT | ACGUGACACGUUCGGAGAATT |
| si-Uqcr10 | CAUAUAAAGCACAAGUAUGTT | CAUACUUGUGCUUUAUAUGTT |
| si-AGO2 | GGUACCACCUGGUGGAUAA | UUAUCCACCAGGUGGUACC |
| si-AGO3 | CUAUGAAGUUGACAUCAAA | UUUGAUGUCAACUUCAUAG |
| si-AGO4 | GCACUUGAUGUGAUUACAA | UUGUAAUCACAUCAAGUGC |
| tRF-Lys-CTT Mimics | GCCCGGCUAGCUCAGUCGGUAGAGCAUGAGACUC |  |
| tRF-Lys-CTT Inhibitor | GAGUCUCAUGCUCUACCGACUGAGCUAGCCGGGC |  |
| Mimics NC | CAGUACUUUUGUGUAGUACAA |  |
| Inhibitor NC | UUGUACUACACAAAAGUACUG |  |

**Table S2.** Primers of real-time qPCR involved in this study.

| **Gene** | **Foward primer** | **Reverse primer** |
| --- | --- | --- |
| METTL1 | CAGACCACACACTGCGCTA | CATCCTTTGGATCATCATGGCTC |
| WDR4 | AGCCGGTTCCTAGCCTTCT | CGTCCTCCCCTTTATCTTCTGG |
| UCP1 | AGGCTTCCAGTACCATTAGGT | CTGAGTGAGGCAAAGCTGATTT |
| β-actin | GTGACGTTGACATCCGTAAAGA | GCCGGACTCATCGTACTCC |
| GAPDH | GAGAGGCCCTATCCCAACTC | TCAAGAGAGTAGGGAGGGCT |
| LPL | GGGAGTTTGGCTCCAGAGTTT | TGTGTCTTCAGGGGTCCTTAG |
| ATGL | CTGAGAATCACCATTCCCACATC | CACAGCATGTAAGGGGGAGA |
| Cidea | TGACATTCATGGGATTGCAGAC | GGCCAGTTGTGATGACTAAGAC |
| COX8b | TGTGGGGATCTCAGCCATAGT | AGTGGGCTAAGACCCATCCTG |
| HSL | CCAGCCTGAGGGCTTACTG | CTCCATTGACTGTGACATCTCG |
| PGC-1α | TATGGAGTGACATAGAGTGTGCT | CCACTTCAATCCACCCAGAAAG |
| tiRNA-1:34-Glu-CTC-1 | TCCCTGGTGGTCTAGTGGTTAGGATTCGGCGCTC | Uni-miR qPCR Primer, included in kit (TaKaRa) |
| tRF-60:76-Tyr-GTA-1-M5 | TCCGGCTCGAAGGACCA | Uni-miR qPCR Primer, included in kit (TaKaRa) |
| tiRNA-1:32-Lys-CTT-3 | GCCCGGCTAGCTCAGTCGGTAGAGCATGAGAC | Uni-miR qPCR Primer, included in kit (TaKaRa) |

**Table S3.** Antibodies involved in this study.

| **Antibody** | **Manufacturer** | **Catalog Number** |
| --- | --- | --- |
| UCP1 | Proteintech | 23673-1-AP |
| METTL1 | Proteintech | 14994-1-AP |
| m7G | MBL | RN017M |
| HSL | Immunoway | YT2240 |
| Uqcr10 | ABclonal | A12587 |
| AGO2 | Abmart | PH1731 |
| Normal Rabbit IgG | Cell Signaling Technology | 2729S |
| Normal Mouse IgG | Santa Cruz Biotechnology | sc-2025 |
| Puromycin | ABclonal | A23031 |
| β-Tubulin | Zenbio | 380628 |
